# A 3D non-human primate digital model for pharmacokinetic prediction of intra-cerebrospinal fluid drug neuraxial dispersion

**DOI:** 10.1186/s12987-025-00723-z

**Published:** 2025-11-04

**Authors:** Mohammadreza Khani, Lucas R. Sass, Ostin Arters, Kathrin Meyer, Bryn A. Martin

**Affiliations:** 1 Neela Therapeutics, Lunenburg, MA USA; 2 Department of Chemical and Biological Engineering, The University of Idaho, Moscow, ID USA

**Keywords:** Cerebrospinal fluid (CSF), Intra-CSF drug delivery, Intrathecal drug delivery, Pharmacokinetics, Computational fluid dynamics (CFD), Non-human primate (NHP), Central nervous system (CNS), OpenFOAM, Digital twin, Craniospinal compliance, Deforming mesh, Solute transport, CSF dynamics, Drug dispersion modeling, In vitro verification, Biofluid mechanics

## Abstract

**Background:**

Intra-cerebrospinal fluid (CSF) drug delivery bypasses the blood-brain barrier, making it a promising route of delivery to treat central nervous system (CNS) diseases. Optimizing this delivery route is challenging because of complex interactions among drug kinetics, CSF flow dynamics and anatomical variations. Non-human primate (NHP) models provide an approximation to human physiology, making a suitable surrogate for studying intra-CSF drug dispersion. We present a NHP digital model for pharmacokinetic prediction of intra-CSF solute neuraxial dispersion that incorporates craniospinal compliance and other key physiological features.

**Methods:**

A 3D subject-specific digital model of the NHP CSF system was formulated using a 3D multi-phase computational fluid dynamics (CFD) approach with flow and geometric boundary conditions using animal-specific in vivo MRI data. Initial digital model drug dispersion predictions were carried out assuming rigid dura and pial surfaces and verified by comparison to a 3D-printed NHP bench-top model replicating the in vivo measurements utilizing fluorescein as a surrogate drug tracer. Once verified, the digital model was extended to mimic craniospinal compliance by incorporating a dynamic mesh to allow dura surface motion that replicated the non-uniform CSF flow along the neuroaxis. Results were quantified over a one-hour period after a 1 mL drug injection via lumbar puncture needle in terms of spatial-temporal drug dispersion along the neuroaxis for the rigid, compliant and bench-top models. Regional percent of injected dose was assessed across the lumbar, thoracic, cervical and cranial regions, while total exposure at each 1 mm section was calculated as the area-under-the-curve (AUC) along the neuroaxis.

**Results:**

The rigid digital model tracer dispersion predictions were verified through comparison with the NHP bench-top model, showing high spatial-temporal agreement (R² = 0.88). The introduction of dynamic mesh motion in the compliant digital model resulted in ~ 10X reduction in peak lumbar CSF flowrate compared with the rigid model (0.065 versus 0.65 mL/min). This decrease in peak CSF flowrate contributed to a reduction in the average Reynolds number along the neuroaxis, dropping from 250 in the rigid model to under 100 in the compliant model leading to decreased tracer dispersion in the lumbar region. At 1 h following injection, tracer distribution to the lumbar, thoracic, cervical and intracranial CSF was 91.9, 8.1, 0 and 0% of injected dose for the compliant model, while a model not including these physiological factors predicted 72.9, 20.4, 5.6 and 1.1%.

**Conclusion:**

The developed NHP-specific digital model, verified with NHP bench-top model simulations, provides a platform to understand and potentially improve intrathecal drug delivery protocols and devices. This study highlights the potentially important role of craniospinal compliance in CSF solute dispersion along the neuroaxis. Incorporating physiological factors such as compliance and varying flowrates into digital models of CSF transport can enhance the predictive capability of drug distribution within the CNS, aiding the design of more effective therapeutic strategies for CNS diseases.

**Clinical trial number:**

Not applicable.

**Supplementary Information:**

The online version contains supplementary material available at 10.1186/s12987-025-00723-z.

## Introduction

 Intra-cerebrospinal fluid (CSF) drug delivery is a promising route of administration for central nervous system (CNS) disease therapeutics because it bypasses the blood-brain barrier. The complex immune structure limits systemic delivery of drugs to the brain through the endothelial tight junctions [1]. Intra-CSF drug delivery is an established route for anesthesia and treatment of chronic conditions including cancer, non-cancer pain, spasticity [2–5] and enzyme replacement or gene therapies for CNS diseases [6]. However, there is a lack of understanding of intra-CSF drug delivery biophysics. Researchers and clinicians have postulated many disparate ideas about how intra-CSF drug delivery can be optimized across numerous parameters of interest. While much in vivo experimental work has been done, it is expensive, lacks consistency and reproducibility and has limited capacity to permutate across the wide delivery optimization landscape [7]. As such, modeling tools are needed to help understand and predict intra-CSF drug delivery biodistribution. As reflected by the growing interest in “digital twins”, digital modeling in tandem with pre-clinical research programs and clinical trials may be employed to inform experimental design, improve drug delivery efficiency, increase target engagement and potentially improve safety.

Prior research has revealed that elucidating optimal intra-CSF drug delivery infusion parameters is not intuitive and consequently may not be effectively accomplished through trial-and-error due to the large number of parameters of interest such as:

- Bolus (buffer) volume, rate, viscosity, density and temperature
- Flush volume, rate, viscosity, density and temperature
- Injection device location, orientation, outlet hole design and customized injection procedures
- CSF formation and clearance rate and location(s)
- CSF flow waveform shape, stroke volume, density and viscosity
- Heart rate, respiratory rate and sleep / circadian cycles
- Impact of injection orientation (gravity) and maneuvers performed during or after injection
- Impact of injection procedures on intracranial pressure, compliance and dynamics
- Altered anatomic and physiological factors due to age and disease state
- Drug-tissue interactions, clearance rate and location(s), molecule size and biochemistry
- Intra-species scaling / allometry for all factors above

Researchers and clinicians have explored how bolus design, flush design, device factors, CSF physiology, posture and gravity, compliance, anatomy, drug properties, and interspecies scaling impact intrathecal dispersion (see Supplementary Table S1 for representative studies). For example, larger bolus volumes have been associated with broader drug dispersion. However, these large volumes may need to be infused at slower rates to mitigate pressure spikes and backflow via the injection device insertion point through the dura​. Additionally, device-related factors such as outlet hole orientation and size may also play a role enhancing neuraxial drug dispersion [8]. Studies have also highlighted the importance of physiologic factors such as variation in CSF dynamics, which is influenced by heart and respiratory rate and characteristics [9]. Long term, aging and disease-related anatomical changes further alter CSF physiology, affecting both drug clearance and distribution [10, 11]. Understanding these factors is a key step in translating findings from animal models to human applications.

An important knowledge gap related to intrathecal drug delivery optimization is a lack of digital (computational) models for prediction of intrathecal solute transport in non-human primates (NHPs). NHP models are considered the gold standard analog for the human CNS and represent a foundational model species in the drug and medical device research and development process. NHPs also allow detailed solute transport validation with dynamic-contrast enhanced MRI or other invasive imaging methods, such as PET and SPECT [12]. However, time and financial limitations prohibit optimization of a large selection of parameters of interest in NHPs. These challenges are driving a push to find alternative model systems [12]. The development of a suitable digital model of CSF drug delivery dynamics in NHPs could offer a rational framework to accelerate and optimize targeting of intra-CSF drug delivery for NHPs and across species to humans.

Prior work with NHP models of intra-CSF drug delivery have demonstrated the utility of computational fluid dynamics (CFD) techniques to simulate CSF flow patterns and biodistribution of intrathecally administered agents. Tangen et al. presented a multiphase digital NHP model of spinal subarachnoid space drug transport [13]. Model predictions of spinal solute transport pharmacokinetics were compared with in vivo measurements using PET and showed similar spatial-temporal dynamics within the spine. Khani et al. formulated a single-phase non-uniform deforming mesh digital model of the NHP spinal CSF flow without nerve roots [14]. This model replicated the non-uniform distribution and waveform characteristics of CSF flow along the spine using subject-specific phase-contrast MR imaging but did not include solute dispersion. Our research team subsequently developed the first laboratory bench-top NHP model of CSF-system wide solute transport and applied it to parametrically investigate the effect of selected lumbar puncture infusion protocols [15]. Comparable parameter sensitivities have also been explored in human-scale computational modeling and imaging investigations [16–25], underscoring the translational continuity between NHP and human models.

While several models of CSF solute transport have been developed in humans and NHPs, there is currently no CSF-system wide digital model of NHP-specific solute transport that includes:

- Key CSF system anatomic regions such as the cortical, intraventricular and basal cisterns of the brain and brainstem, which are required to recapitulate regional solute dispersion characteristics.
- A complete spinal subarachnoid space with anatomically detailed individual dorsal and ventral spinal cord nerve rootlets, radicular line, descending angle, and filum terminale features to capture complex nerve root-related mixing effects along the spine.
- Non-uniform distribution of neuraxial compliance that reproduces the physiological attenuation of CSF stroke volume along the spine and around the brain to allow solute dispersion within the entire NHP CSF system.
- Inclusion of cardiac- and respiratory-induced CSF oscillations in the spine and aqueduct of Sylvius to capture potential mixing effects due to respiration and within the ventricular spaces of the brain.
- Inclusion of CSF production and absorption from the choroid plexus and arachnoid granulations in the model to allow dilution and outflow effects on intracranial solute dispersion.

Here we describe our novel model system addressing these significant challenges.

## Methods

**Fig. 1:**
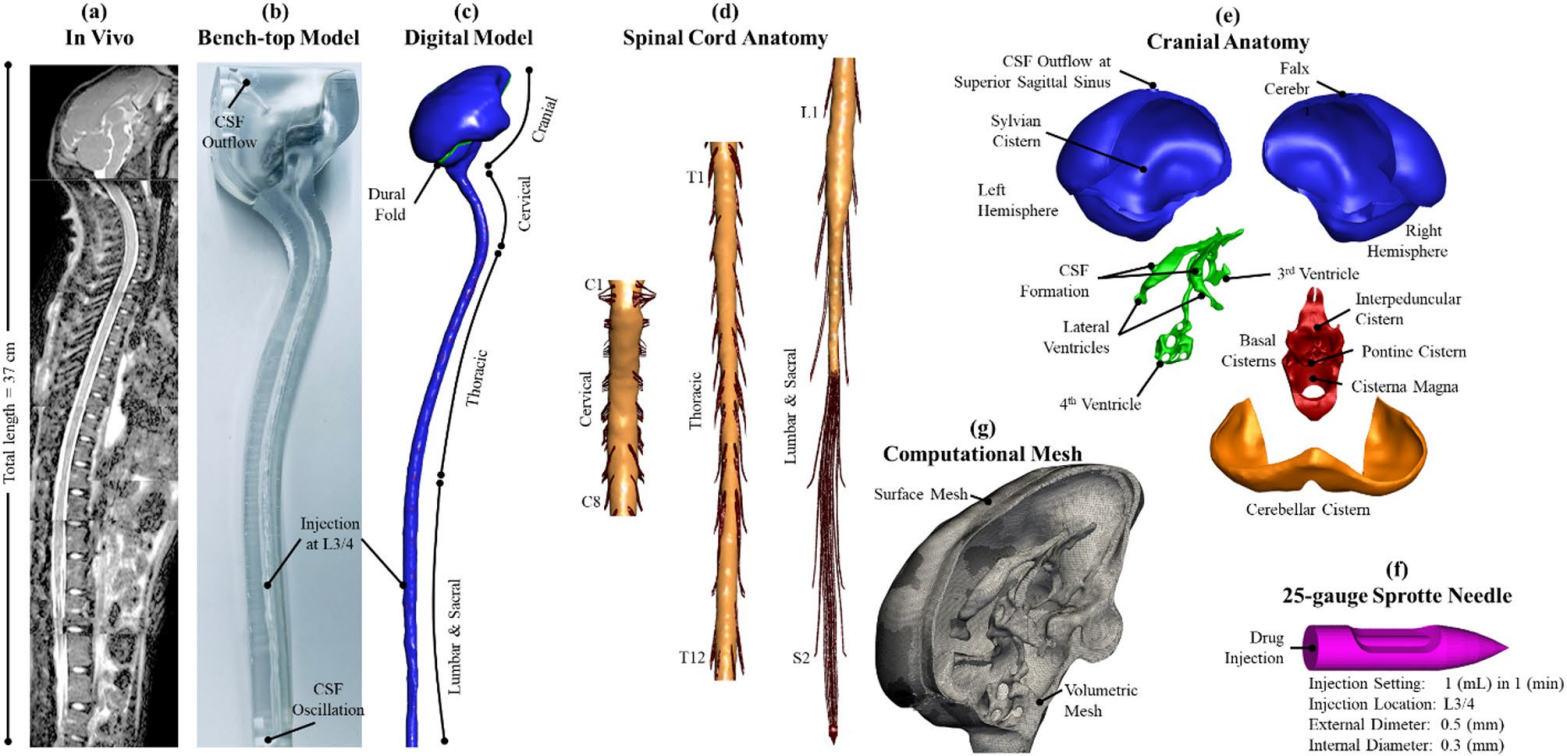
Overview of numerical and bench-top model using animal-specific MRI measurements. (**a**) T2-weighted MR image used to acquire subject-specific anatomy and natural CSF oscillation. (**b**) 3D-printed bench-top model designed identical to numerical model for verification (**c**) Computational model of the entire CSF space for NHPs (**d**) Magnification of cervical, thoracic, lumbar and sacral nerve roots (**e**) Magnification of cranial subarachnoid space compartments consisting of ventricular system, cerebellar cistern, basal cisterns and cortical subarachnoid space (left and right) (**f**) Visualization of the 25-gauge spinal needle 3D-model used for tracer injection. (**g**) Visualization of surface mesh and cut-away volume mesh illustrating finely graded cells in surrounding ventricular and cranial cistern regions

The following procedures were applied to extract CSF geometry and flow from MRI data collected for a 4-year-old, male, 4.0 kg, cynomolgus macaque NHP. Subsequently, these data were employed to construct animal-specific 3D models to test system wide solute transport using in vitro and digital modeling techniques (Fig. 1a-c).

### NHP imaging protocol

CSF space geometry and flow were collected using the protocol described by Khani et al. [26] on a Philips 3T MRI scanner (ACHIEVA, software V2.6.3.7, Best, The Netherlands) with a total scan time of 81 min. To define CSF space anatomy, including spine, brain and ventricles, a stack of 720 axial images was collected using a volumetric T2-weighted acquisition (VISTA) reconstructed with 0.5 mm slice spacing and 0.38 mm isotropic in-plane resolution (Fig. 1a).

### Animal-specific spinal subarachnoid space and nerve root geometry

The spinal subarachnoid space was manually segmented from the T2-weighted images using ITK-SNAP (Version 3.0.0, University of Pennsylvania, U.S.A.). A single representative animal, NHP-01, was selected using a review of factors including body weight, age, and rostro-caudal length. Animal-specific detail for the spinal cord nerve roots was not possible to directly visualize. Thus, an idealized nerve root geometry previously developed by our group for a human subject was adapted to the NHP spine due to the strong anatomic similarity across species, including 31 nerve rootlets pairs and other factors [27]. The T2-weighted MR images were reviewed manually to identify vertebral intersections and dorsal root ganglion locations along the spine. Human nerve root geometry was then scaled linearly using the ratio of NHP to Human spinal-canal length below the foramen magnum. The 61.5 cm human value comes from the MRI dataset used in our earlier human CSF model and falls within the 60–65 cm adult range [28], whereas the 30.1 cm macaque length was measured in the present images, together justifying the 0.5 scaling applied to the root lattice. Because individual macaque rootlets (< 0.25 mm) are smaller than the 0.38 mm imaging voxel, we down-scaled the validated human root lattice by the same 0.50 factor to preserve the root-to-canal area ratio in the NHP spine. Individual dorsal and ventral nerve root placement was then performed by manual rigid transformations in Blender (Blender Foundation, version 4.4) such that the intersection of each nerve root pair with the dura occurred at the corresponding landmark for the dorsal root ganglion (Fig. 1d). For the cervicothoracic nerve roots, these rigid transformations were done independently for the left and right lateral pairs of anterior and posterior rootlets to ensure that these pairs intersected both the spinal cord and dorsal root ganglion intersections. Below the spinal cord conus, the lumbosacral nerve roots become elongated and parallel which necessitated a remodeling of their trajectories to match the unique spinal canal curvature in the specific NHP. These individual rootlet trajectories were redefined by extending a path from the conus to the corresponding lumbosacral dorsal root ganglion. A cylindrical extrusion was then defined along these paths with a rootlet diameter applied using our previous study with a scaling factor of 0.5 as detailed in Sass et al. [29].

### Animal-specific cranial subarachnoid space and ventricular system geometry

An animal-specific manual segmentation was performed by a single expert operator in ITK-SNAP to define the intracranial dural and pial surface of each cerebral hemisphere, cerebellum, and brainstem separately including extra-axial CSF, Sylvian cisterns, cisterna magna, and basal cisterns of the brain (Fig. 1e). Segmentation of the intraventricular spaces included the lateral, third and fourth ventricles, and aqueduct of Sylvius. Post-processing of these segmentations was subsequently performed in Blender. Each segmented structure was imported with a Laplacian smoothing modifier applied to reduce surface noise. For some locations around the brain, extra-axial CSF space thickness was smaller than the imaging resolution and a minimum arachnoid-pial distance of 50 μm was assumed to maintain continuous surface separation for both computational and 3D-printed bench-top model applications. The 3-D printed phantom geometry was segmented directly from the T2-weighted MRI dataset described in Sect. 2.1, ensuring a one-to-one correspondence between the benchtop model and the numerical domain. Care was taken to ensure that changes to regional CSF space volumes and anatomic detail were retained. Where tiny sulcal pockets were smaller than voxel size, we slightly offset the local segmentation boundary outward so that Laplacian smoothing did not change the total intracranial CSF volume. After smoothing, a series of Boolean operations were then applied between these smoothed surfaces to reconstruct an intracranial CSF system consisting of the extra-axial CSF, cerebellar CSF, ventricular system, and basal cisterns of the brain. The intracranial model was combined with the spinal model to form a complete 3D NHP CSF geometry.

### 3D NHP computational mesh creation

Using the 3D geometry, a computational mesh was formulated to represent a standard intra-CSF drug delivery procedure via lumbar puncture (LP) using a standard 25-gauge Sprotte needle inserted at L3/4 vertebral level. The outer and inner needle diameter was 0.5 mm and 0.3 mm, respectively (Fig. 1f). The Snappy Hex Mesh utility, part of the OpenFOAM (Open Field Operation and Manipulation) software package, was utilized to generate the computational mesh. Initially, a coarse hexahedral block mesh with resolution of 50*60*200 along x, y, and z direction was created to encapsulate the entire CSF domain. Surface features with angles lower than 150 degrees were extracted from the geometry’s surface, defined by STL files. Subsequently, the mesh around these extracted surface features was adaptively refined by SnappyHexMesh by snapping the mesh vertices onto the surface. The final mesh included a combination of hexahedral and polyhedral elements alongside triangular and quadrilateral surface meshes for capturing fine surface details. Mesh density varied from 80,000 to 160,000 cells/mL, with approximately 2.1 million total cells being generated for the entire CSF system to achieve mesh-independent results. The internal needle mesh was refined to resolve the inlet velocity profile and drug kinetics during infusion. Special attention was given to improving mesh quality, particularly around the injection site, nerve roots and ventricular system by iterative smoothing and refinement until the maximum face skewness was maintained below 1 and the cell growth aspect ratio did not exceed 1.2. Region-based refinement was then applied so that both large cavities and the narrow 0.8 mm aqueduct were resolved at the correct scale. Ventricular surfaces were flagged for level 3 refinement, giving 0.20–0.25 mm cells, whereas a 1 mm-radius cylindrical zone along the aqueduct centerline was set to level 5, forcing a 0.05 mm edge. A maxGrowth limit of 1.2 prevented abrupt jumps between the 0.05 mm aqueduct cells and the coarser ventricular grid, and all final elements satisfied non-orthogonality < 60° and skew < 1.

### CSF flowrate

**Fig. 2:**
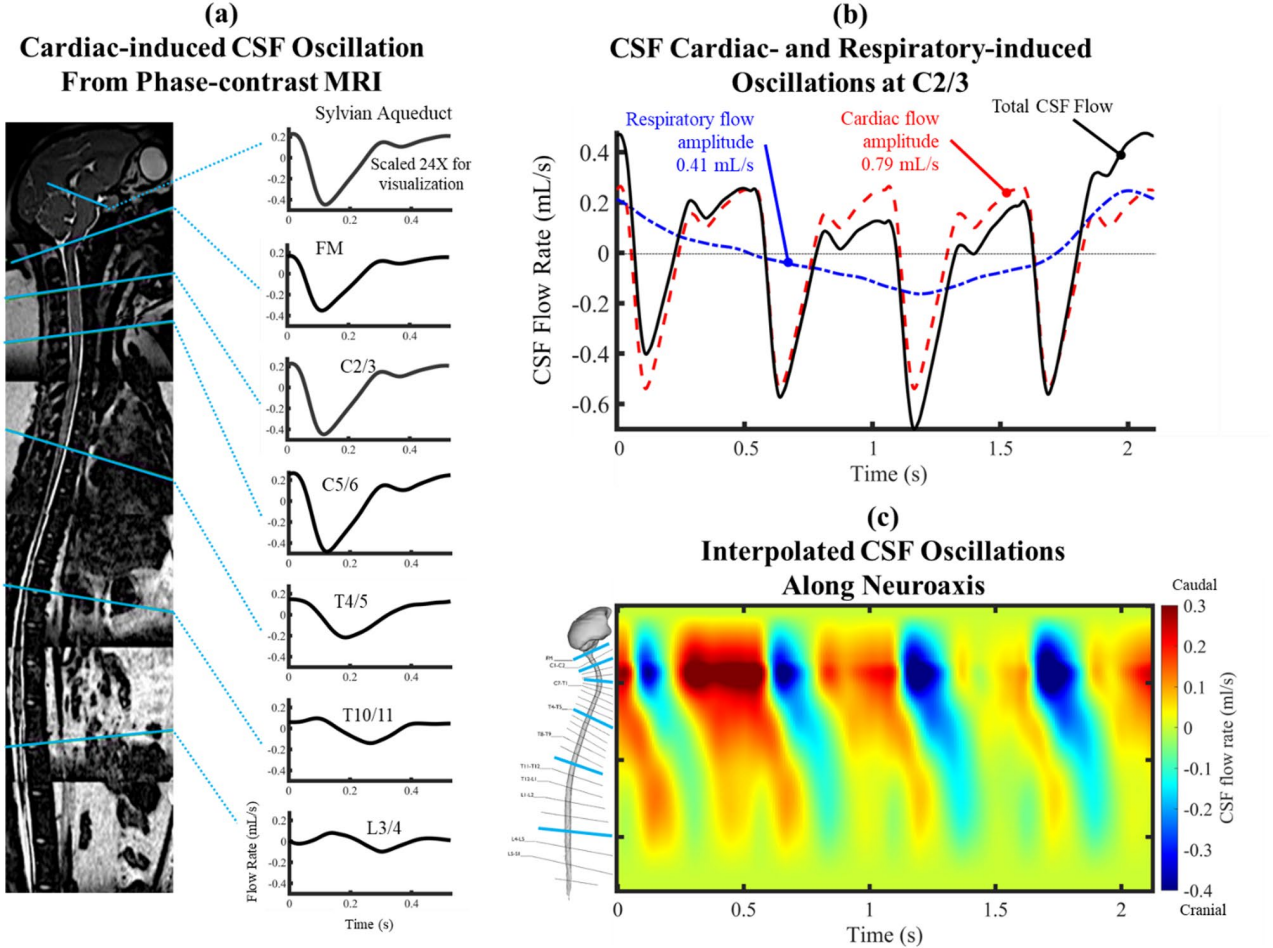
Uniform CSF flowrate for rigid digital and bench-top model and non-unform CSF flowrate for compliant digital model. (**a**) The CSF flowrate based on in vivo PCMRI measurement at the foramen magnum, C2-3, C5-6, T4-5, T10-11 and L3/4. (**b**) Cardiac CSF flowrate at C2/3 superimposed on respiratory based CSF flowrate using natural respiration from Yildiz et al. with Red = cardiac CSF waveform (115 bpm); blue = respiratory waveform (28 bpm, amplitude = 0.52 × cardiac); black = cardiac + respiratory sum. Four cardiac cycles occur within each respiratory cycle. Dashed line indicates zero flow. (**c**) Spatial-temporal distribution of the interpolated CSF flowrate along the spine for the compliant digital model

For CSF flow quantification, phase-contrast MRI measurements were collected with retrospective electrocardiogram (ECG) gating with 24 heart phases reconstructed over the cardiac cycle with 0.45 × 0.45 mm isotropic in-plane reconstructed resolution and 5 mm slice thickness based on our prior research [26, 30]. The slice location for each scan was perpendicular to the CSF flow direction along the neuroaxis with slice planes intersecting vertebral disks. The locations included the foramen magnum and vertebral disks located at the C2/3, C5/6, T4/5, T10/11, and L3/4 vertebral levels (Fig. 2a). Velocity encoding was set to 5 cm/s at the foramen magnum and L3/4 and 10 cm/s at all other axial locations. Velocity encoding values were selected based on expected peak CSF velocities at each level from prior studies. Mid-spinal levels exhibit peak velocities up to ~ 8 cm/s, prompting the use of VENC = 10 cm/s to prevent aliasing. Lower velocities are observed near the foramen magnum and L3/4 (~ 3 cm/s) where a VENC = 5 cm/s setting improved resolution while limiting signal noise. These parameters are consistent with our previous NHP flow imaging work [31].

After acquiring cardiac-based CSF flowrates from MRI, respiratory-based CSF flowrates were incorporated to simulate natural respiration in an NHP using the method previously described by Burla et al. [15]. In brief, the human respiratory-induced CSF flow waveform shape quantified by Yildiz et al. [32] was applied for the NHP digital model where the respiratory to cardiac amplitude ratio of 0.52 was assumed to be preserved across species. Also, NHP average respiratory rate applied by Burla et al. was 28 breaths per minute and cardiac-induced CSF oscillation rate was 115 beats per minute leading to a cardiac-to-respiratory rate ratio of ~ 4.10. This ratio was rounded down to 4.0 to mitigate aliasing of optical tracers in the bench-top model. The phase- and amplitude-scaled cardiac and respiratory CSF flowrates were then superimposed. This process was repeated for each phase-contrast MRI measurement location, with the C2/3 CSF flowrate serving as the primary CSF oscillation in the rigid bench-top and rigid digital model (Fig. 2b). For the compliant digital model, the spatial-temporal CSF flow distribution along the neuroaxis was determined by interpolating the CSF flowrates across the six vertebral levels (Fig. 2c). Also, CSF flow was assumed to attenuate to zero at the spinal termination and at the intracranial vertex near the superior sagittal sinus. Similar to Burla et al. [15], the CSF flow waveform at the aqueduct of Sylvius was scaled with a 1 to 24 ratio of the CSF flowrate at the C2/3 level, with a 25 mL/day net flow representing NHP CSF formation rate measured in vivo by McCully et al. [33]. In the absence of direct PC-MRI data from the aqueduct, we imposed a scaled version of the C2/3 flow waveform with reduced amplitude. While this does not capture potential time lags specific to the aqueduct, the flow amplitude in this region is minimal relative to spinal flow and net CSF production from the ventricles limits anterograde solute dispersion [34, 35]. Thus, the NHP aqueductal CSF flow waveform assumption is expected to have little impact on overall solute dispersion results.

### Deforming mesh method for the compliant model

Mesh deformation was computed using the interpolated CSF flow rate along the neuroaxis as described previously [14] and extended to attenuate CSF flow to zero between the foramen magnum and superior sagittal sinus. The radial displacement of the dura ($\:{\triangle\:r}_{(z,t)}$) for each section along the spinal axis (z) and time step (t) was computed as:

1 $$\:\:\:{\triangle\:r}_{(z,t)}=\:\sqrt{{r}_{\left(z\right)}^{2}-\frac{{\triangle\:Q}_{(z,t)}\times\:\triangle\:t}{\pi\:\times\:h}}-{r}_{\left(z\right)}$$

**Fig. 3:**
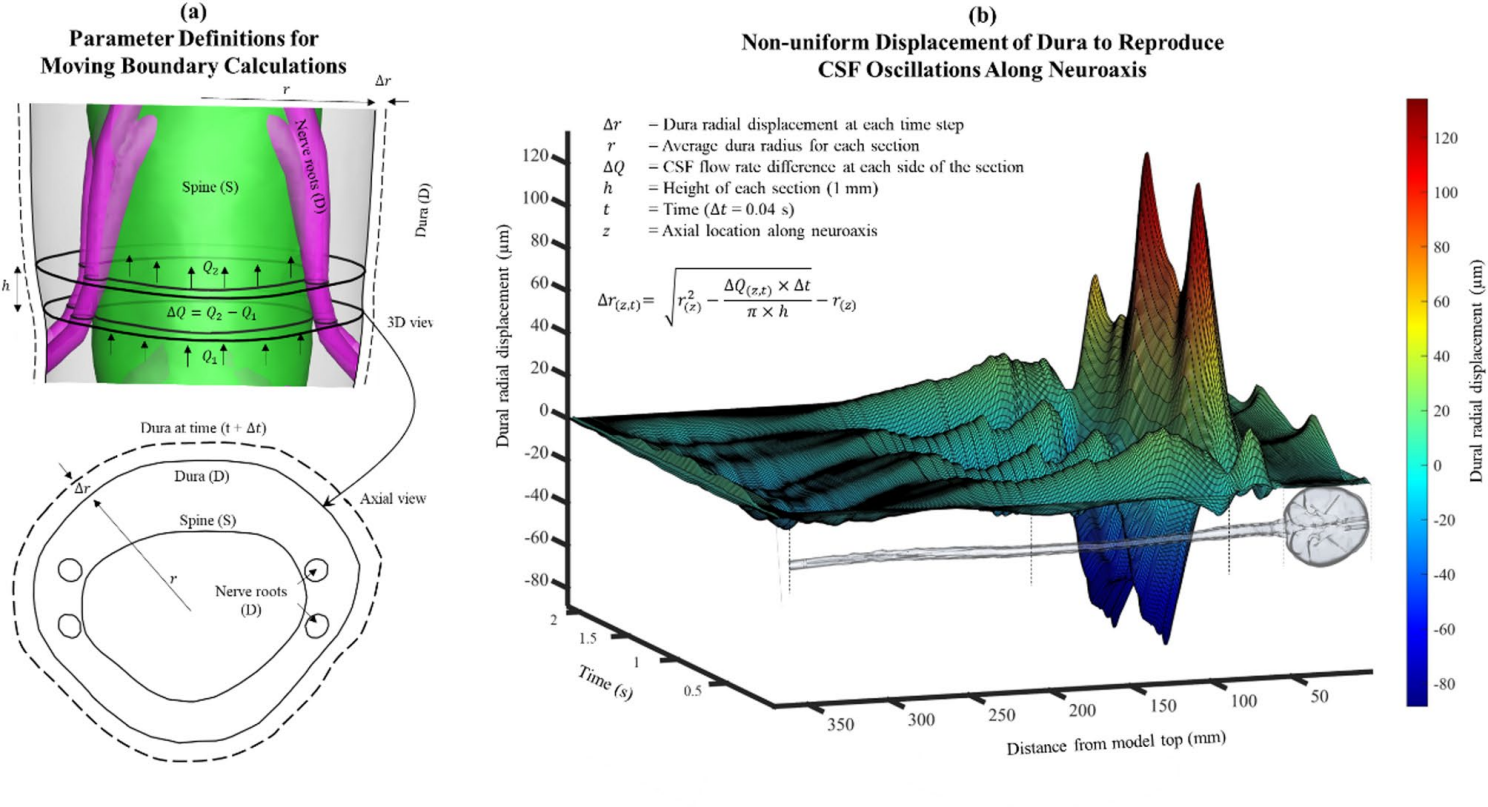
Implementation of deforming mesh motion algorithm in CFD simulations: (**a**) Depicts both a 3D representation and an axial view of a typical section utilized for the computation of radial deformation. (**b**) Illustration of spatial-temporal distribution of dura radial displacement along the spinal column and radial deformation equation

Where, $\:{\triangle\:Q}_{(z,t)}$ is the difference between CSF flowrate on either side of a given section at each time step (Fig. 3a), $\:{r}_{\left(z\right)}$is the section radius, and h is the section height assumed to be uniform at 1 mm for all sections. Figure 3b shows the calculated spatial-temporal mesh movement radially, $\:{\triangle\:r}_{(z,t)}$, for each 1 mm section that was imposed on the dura surface at each time step to replicate the flowrate at each section.

### Computational solver and boundary conditions

Numerical simulation of solute dispersion within the oscillatory CSF flow field was conducted using the laminar mixture multiphase model derived from driftFluxFoam within OpenFOAM. The drift coefficients were set to zero due to CSF Newtonian fluid behavior. The dynamic mesh solver from InterIsoFoam was incorporated into the solver to allow non-uniform dynamic mesh motion. A variable time step size, with a maximum of 0.001 (s) during injection and 0.01 (s) post-injection, was used to maintain a maximum Courant number < 0.5. This temporal refinement allowed to capture the transient drug dispersion behavior from the needle tip. Convergence criteria for velocity and pressure were set to 1e-7 with a relative tolerance of 0.01. Given the oscillatory nature of CSF flow, momentum prediction was deactivated to enhance convergence with relaxation factors set to 1.

Like our previous human digital multiphase simulations, tracer diffusivity was omitted from the model physics [34]. CSF formation and clearance rate was specified to be 25 mL/day [36] and located at the choroid plexus and superior sagittal sinus, respectively. A zero gradient outlet boundary condition was specified at the superior sagittal sinus (Fig. 1b). A non-slip boundary condition was specified at all other surfaces including the spinal cord, dura, brain, ventricles and nerve roots. CSF and the injected drug solution were assumed to be incompressible with a density of 1000.59 kg/m³ and dynamic viscosity of 0.69 mPa⋅s similar to CSF at body temperature [37]. A standard NHP lumbar puncture injection procedure was simulated with a neutrally buoyant 1 mL drug injection at a rate of 1 mL/min.

### Bench-top model

To verify the rigid NHP digital model results, a bench-top model with an identical 3D-printed fluid geometry and flow boundary conditions was constructed and tested (Fig. 1b) as described in Burla et al. [15] and additional imaging methods detail in Seiner et al. [38]. In brief, the rigid CSF phantom was 3D-printed (Stratasys Direct MFG) using optically transparent Somos^®^ WaterShed XC 11,122. Deionized water was utilized as a CSF surrogate and fluorescein sodium (fluorescein) was used as an optical tracer in the infusion bolus [39]. To visualize tracer dispersion, a custom designed optical imaging system was calibrated using reference data sets collected under a range of known uniform fluorescein concentrations. Calibrated data sets allowed quantification of pixel-by-pixel concentration of tracer from two projection angles which were averaged to obtain the bench-top spatial-temporal tracer distribution at each imaging time point. A pump-driven, time-varying inlet-flow waveform measured by MRI at the C2/C3 vertebral level, identical to the rigid CFD boundary condition, was applied at the sacral end of the phantom. In the compliant CFD model no inlet or outlet flow boundaries were imposed; craniospinal motion was generated solely by slice-wise radial dura displacements derived from the PC-MRI flow gradient. Oscillatory CSF flow input to the bench-top model was verified by an inline Transonic precision PXN-series flow sensor (Transonic System, Ithaca, NY). The root mean square error (RMSE) was computed between the combined cardiac and respiratory ground truth waveform derived from in-vivo MRI flow versus the mean experimental flow measured over 100 cycles in the bench-top model.

### Geometric and hydrodynamic quantification

**Fig. 4:**
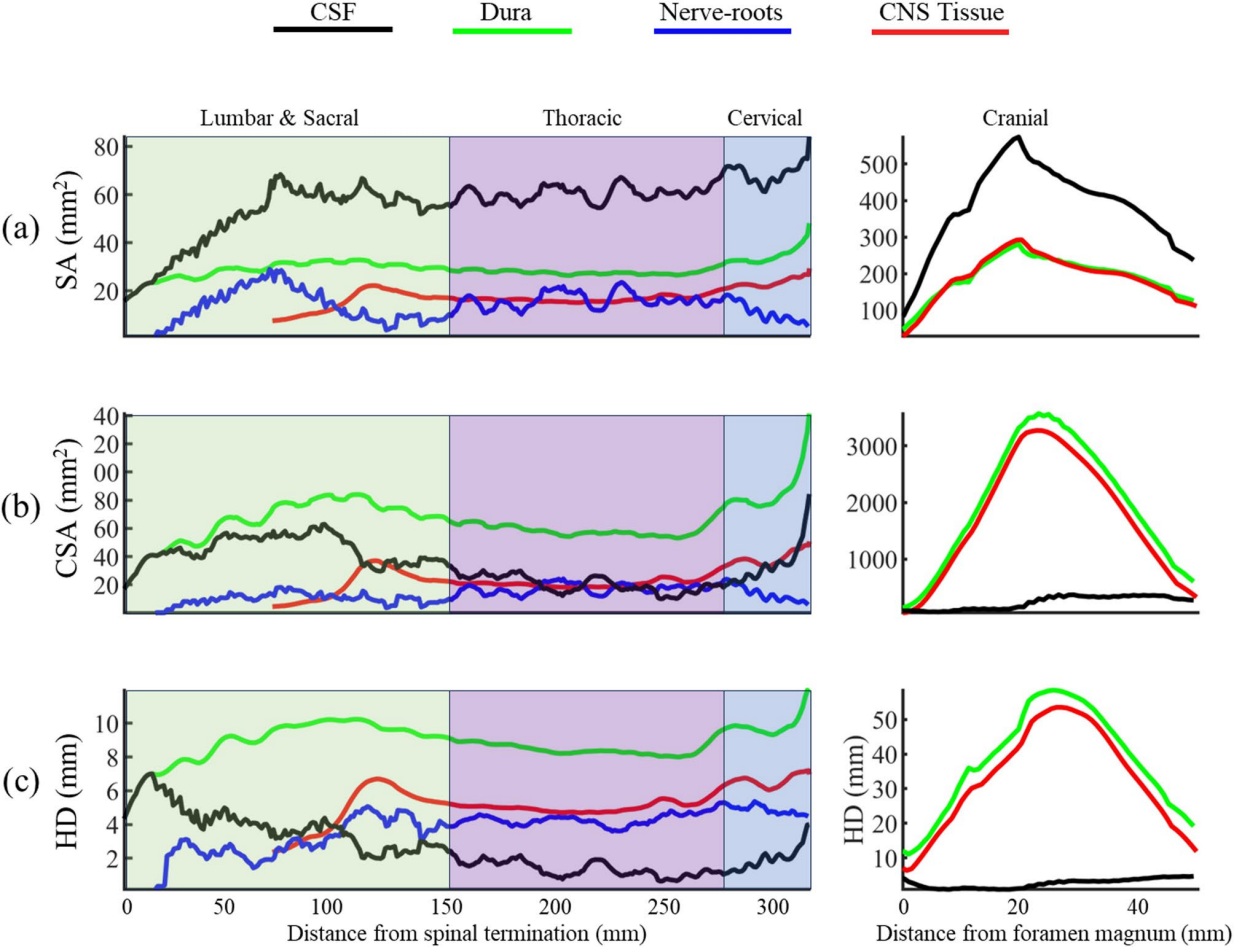
Distribution of geometric parameters computed based on 1 mm slice sections across anatomical regions including the dura mater, spinal cord, nerve roots, and subarachnoid space along the spinal and cranial regions of a NHP. The parameters visualized include (**a**) surface area (SA), (**b**) cross-sectional area (CSA), and (**c**) hydraulic diameter (HD)

We quantified the following geometric and hydrodynamic parameters for the NHP model [34, 38]. Geometric parameters included the surface area (SA), cross-sectional area (CSA), and hydraulic diameter (HD) for the dura, CNS tissue (including brain and spinal cord), nerve roots, and CSF space within each 1 mm slice interval along the neuroaxis (Fig. 4). The hydraulic diameter was calculated as: $\:HD=4\frac{CSA}{{P}_{w}}$ where $\:{P}_{w}$ is the wetted perimeter measured at each slice. Hydrodynamic parameters included peak CSF flowrate (*Q*_*peak*_), mean peak CSF velocity ($\:{U}_{peak}=\frac{{Q}_{peak}}{CSA}$), and maximum Reynolds number ($\:Re=\frac{\rho\:{U}_{peak}HD}{\mu\:}$) (Fig. 8), where *ρ* is CSF density and *µ* is CSF dynamic viscosity.

### Results visualization and post-processing

Velocity contours were visualized at 6 different slices along the spine for both rigid and compliant digital models. 3D tracer concentration was visualized within the spine and brain for selected time points after infusion. Concentration values are presented as the percentage of CSF volume that is occupied by the tracer. Additionally, spatial-temporal tracer evolution along the neuroaxis and brain was quantified in terms of the cross-sectional average tracer concentration up to 1-hour post-injection. Regional tracer mass was calculated as a percentage of the injected drug bolus for the cranial and spinal subarachnoid space (lumbar, thoracic and cervical) at 1-hour post-injection where$\:\:Percent\:of\:injected\:dose\:\left(\%\right)=concentration\:\left(\%\right)$ $*\frac{region\:volume\:\left(mL\right)}{bolus\:volume\:\left(mL\right)}$. Area under the curve, $\:{AUC}_{\left(z\right)}$, was calculated as the summation of the percent injected dose within each 1 mm slice thickness along the neuroaxis at 1 (s) intervals over 1-hour (%·hr).

### Bench-top verification of digital model results

To help verify digital model predictions, the rigid digital model results were compared to the 3D-printed bench-top model with identical geometry and flow boundary conditions. Model agreement was quantified using linear regression and Bland-Altman analysis of the 1-hour spatial temporal mass distribution for the rigid digital versus bench-top model [34].

## Results

### NHP model geometry

Figure 1 shows the final NHP model geometry along with a detailed view of the 31 pairs of nerve rootlets (Fig. 1d) and intracranial CSF spaces (Fig. 1e). The cynomolgus NHP 4th ventricle was noted to have two additional foramina allowing CSF to exit the 4th ventricle with five openings, unlike the human 4th ventricle with three (see Fig. 1e – 4th ventricle region). Surface areas for the spinal cord (CNS contacting) and dura within the spinal canal was 47.0 cm^2^ and 94.8 cm^2^, respectively. Surface area for all spinal cord nerve rootlets totaled 22.7 cm^2^ with a 4.3 cm^2^, 10.5 cm^2^ and 7.8 cm^2^ split between the cervical, thoracic and lumbosacral regions. Nerve rootlet diameter ranged from 0.25 mm to 0.55 mm with a mean of 2.25 rootlets per-side, per-vertebral level (see rootlet details in Table 1).

**Table 1:** Anatomic measurements obtained from the 3D NHP spine including the dorsal and ventral rootlet descending angle, radicular line, diameter, and number of rootlets

		Dorsal Rootlet Measurements (Average of Left and Right)				Ventral Rootlet Measurements (Average of Left and Right)			
Nerve Root #	Z Position	Descending Angle	Radicular Line	Diameter (mm)	Number of Rootlets	Descending Angle	Radicular Line	Diameter	Number of Rootlets
	(mm from foramen magnum )	° below horizontal	mm	mm	n	° below horizontal	mm	mm	n
C1	3.6	4.6	3.0	0.35	4	5.1	3.2	0.35	3
C2	7.6	5.9	2.7	0.35	4	6.2	4.0	0.35	4
C3	12.5	35.9	4.5	0.35	6	28.3	4.9	0.35	5
C4	19.8	40.4	3.9	0.4	5	37.0	3.6	0.4	5
C5	26.0	37.8	3.3	0.4	5	37.3	3.1	0.4	5
C6	31.1	55.2	4.8	0.45	4	39.5	5.1	0.45	4
C7	36.5	71.5	2.9	0.5	4	59.6	3.0	0.5	4
C8	44.4	67.2	1.6	0.45	2	58.6	1.9	0.45	2
T1	50.7	68.0	3.2	0.55	3	64.3	3.3	0.55	3
T2	59.1	70.3	2.7	0.55	2	69.9	2.7	0.55	2
T3	69.3	75.0	3.9	0.55	2	68.6	4.0	0.55	2
T4	77.6	68.5	4.2	0.55	2	65.8	4.1	0.55	2
T5	81.8	74.4	3.3	0.55	2	73.1	3.0	0.55	2
T6	93.7	76.1	3.2	0.55	2	76.2	3.3	0.55	2
T7	100.3	79.5	4.3	0.55	2	78.7	4.3	0.55	2
T8	109.1	71.3	3.9	0.55	2	79.8	3.8	0.55	2
T9	118.4	79.9	3.3	0.55	2	79.7	3.0	0.55	2
T10	130.4	78.6	4.3	0.55	2	77.7	6.0	0.55	2
T11	147.3	79.8	9.5	0.55	2	77.4	10.0	0.55	2
T12	166.3	75.7	4.2	0.55	1	74.8	4.1	0.55	1
L1	181.3	74.6	4.8	0.55	1	82.6	4.5	0.55	1
L2	193.5	77.0	2.8	0.55	1	85.1	3.0	0.55	1
L3	208.8	87.1	2.0	0.75	1	88.0	2.0	0.75	1
L4	214.1	87.0	0.7	0.65	1	87.6	0.7	0.65	1
L5	218.9	87.3	0.6	0.6	1	86.9	0.6	0.6	1
S1	221.2	86.9	0.5	0.5	1	87.0	0.5	0.5	1
S2	224.7	86.6	0.3	0.25	1	87.5	0.3	0.25	1
S3	230.8	86.6	0.3	0.25	1	87.0	0.3	0.25	1
S4	230.8	86.8	0.3	0.25	1	87.3	0.3	0.25	1
S5	230.8	87.3	0.3	0.25	1	87.5	0.3	0.25	1

Total CSF volume was 16.2 mL (Table 2), with 7.8 mL in the spine and 8.3 mL in the cranial vault. Volume of the spinal cord and nerve roots was 3.9 cm^3^ with 0.6 cm^3^, 1.9 cm^3^, and 1.4 cm^3^ split between cervical, thoracic, and lumbosacral region, respectively. 70% of the intracranial CSF volume was contained in the cortical subarachnoid space with a slight asymmetry of 2.8 mL and 3.1 mL around the right and left hemispheres, respectively. Only 6% of the cranial CSF (0.53 mL) was contained in the ventricular system including the lateral ventricles, third ventricle, aqueduct of Sylvius and fourth ventricle. The cerebellar CSF spaces including the cisterna magna contributed 0.39 mL or 2% with the remainder, 1.59 mL or 10%, located in the basal cisterns of the brain.

**Table 2:** Summary of geometric parameters related to CSF-tissue interfaces within the digital and bench-top model

Region	CSF Volume (mL)	CSF Surface Area in Contact with Dura (cm^2^)	CSF Surface Area in Contact with CNS tissue (cm^2^)	Spinal Cord Nerve Rootlet Volume (cm^3^)
Lumbar	3.60	42.0	11.9	1.4
Thoracic	2.75	36.4	23.4	1.9
Cervical	1.45	16.4	11.7	0.6
**Total**	**7.80**	**94.8**	**47.0**	**3.9**
Left hemisphere	2.79	38.9	37.7	N/A
Right hemisphere	3.13	41.3	38.8	N/A
Cerebellum	0.39	7.2	7.2	N/A
Basal cisterns	1.59	8.9	16.4	N/A
Ventricular system	0.53	N/A	11.2	N/A
**Total**	**8.43**	**96.3**	**111.4**	N/A
**Grand Total**	**16.23**	**191.1**	**158.4**	

Average CSF cross-sectional area (CSA) in the spine was ~ 60 mm² (Figs. 4a-b), which is about five times smaller than the intracranial average CSA of ~ 300 mm². Hydraulic diameter (HD) was similar in the spinal and intracranial subarachnoid space, with an average value of 2 mm throughout the model (Fig. 4c).

### Verification of rigid digital model by bench-top experiments

**Fig. 5:**
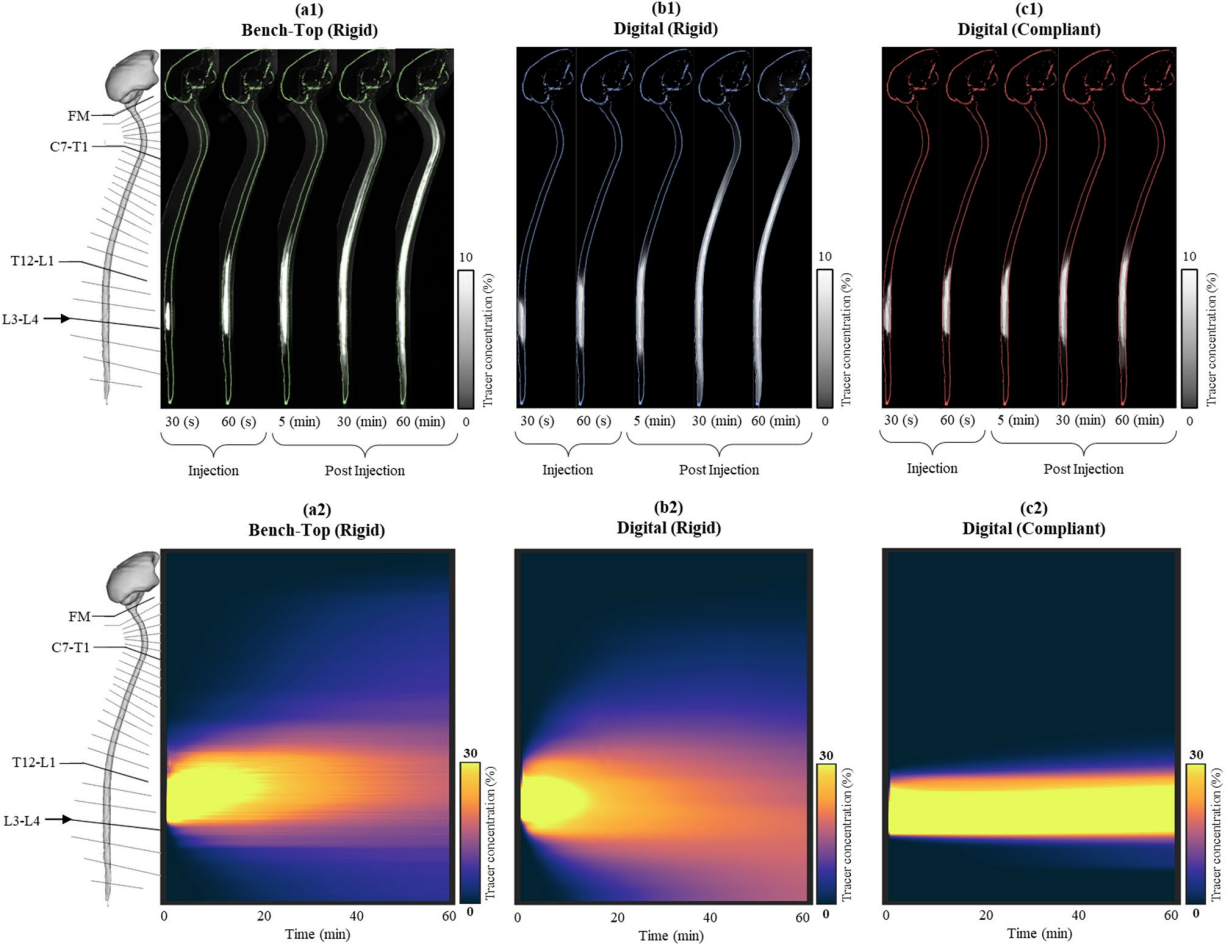
3D representation of tracer concentration and spatial-temporal percent injected dose over 1-hour for the (**a**) rigid bench-top model, (**b**) rigid digital model, and (**c**) compliant digital model. The visualization is further subdivided to depict tracer concentration during the injection period of one minute and then over the subsequent post-injection period of 1-hour

**Fig. 6:**
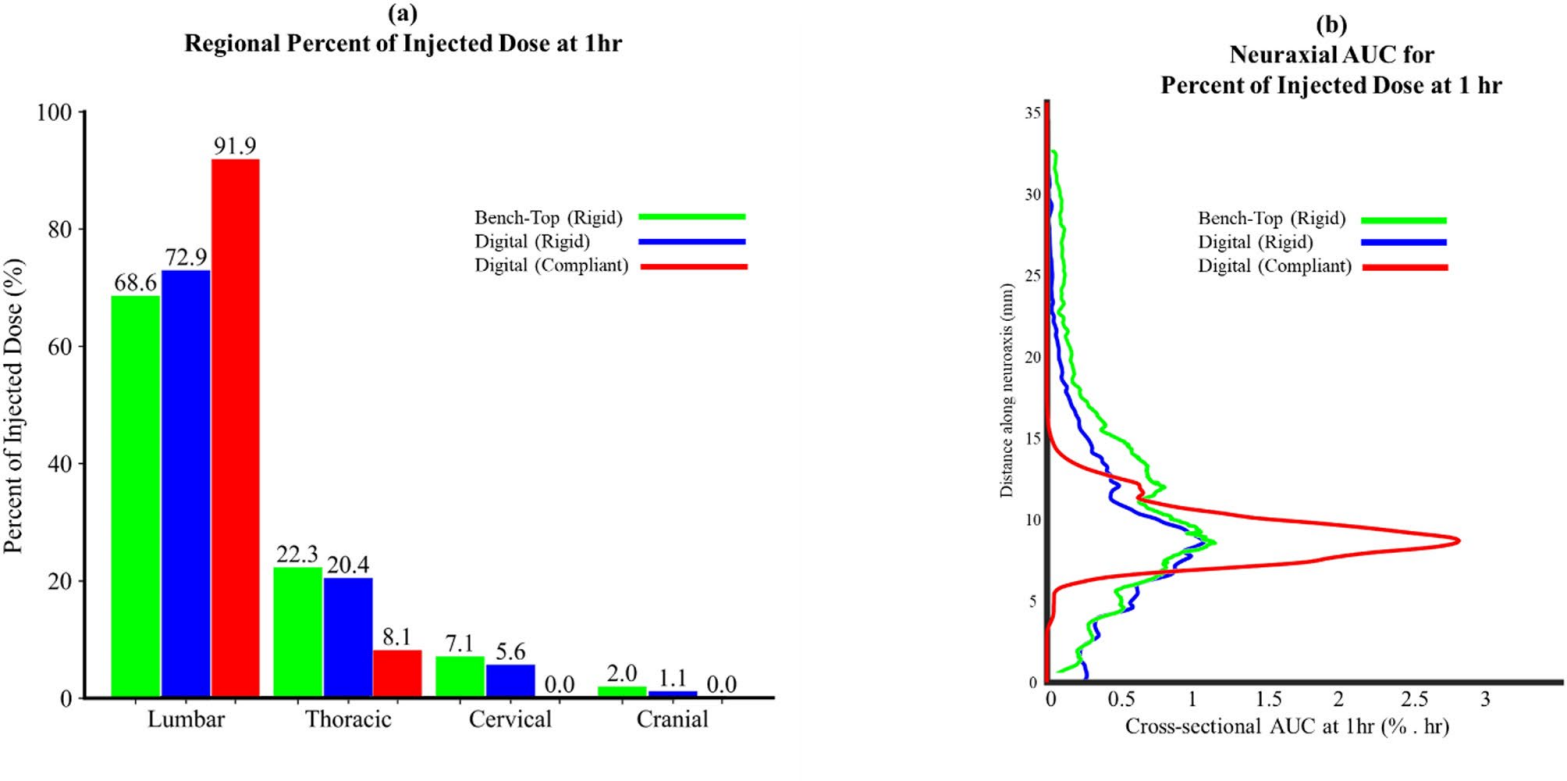
Summary of regional percent injected dose and AUC values for the bench-top and digital models. (**a**) Bar plot showing the percent injected dose in different regions: lumbar, thoracic, cervical, and cranial. (**b**) Area under the curve (AUC) of the tracer at each 1 mm slice over a 1-hour period following injection

Rigid digital model predictions were strongly correlated with the bench-top model experiments. Bland-Altman statistical analysis of the 1-hour spatial temporal mass distribution for the rigid digital and bench-top models showed an R² value of 0.88, slope = 1.07 and 95% confidence interval of 8.9% of the injected dose. Tracer in both models reached the T11 level by the end of the injection at 60 s (compare Fig. 5a1 and b1). Both models had a peak tracer concentration of approximately 30% near the injection site during the first 20 min post infusion followed by a gradual decline (as shown in Fig. 5a2 and b2). At 60 min post-injection, regional mass distribution (Fig. 6a) and AUC profiles (Fig. 6b) showed strong similarity.

### Impact of CSF system compliance

**Fig. 7:**
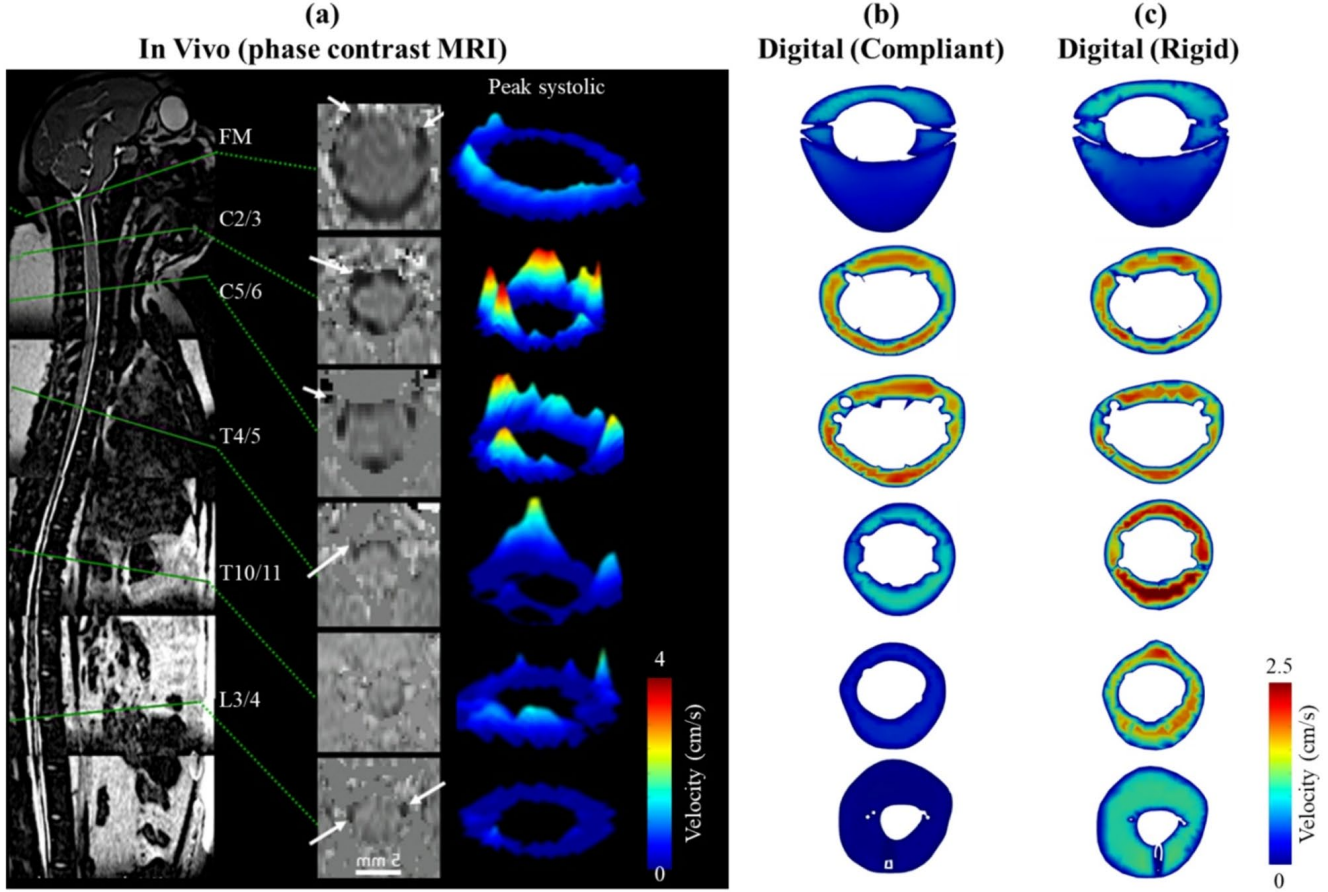
Agreement of in vivo MRI velocity field compared to the compliant and rigid digital model. (**a**) In vivo depiction of NHP T2-weighted MRI mid-sagittal view with axial phase contrast MRI peak CSF velocity distribution around the spinal cord and 3D velocity field visualization (image modified from [30]), (**b**) Compliant digital model velocity profile at foramen magnum, C2/3, C5/6, T4/5, T10/11 and L3/4 vertebral levels, and (**c**) Rigid digital model velocity profiles at same locations

**Fig. 8:**
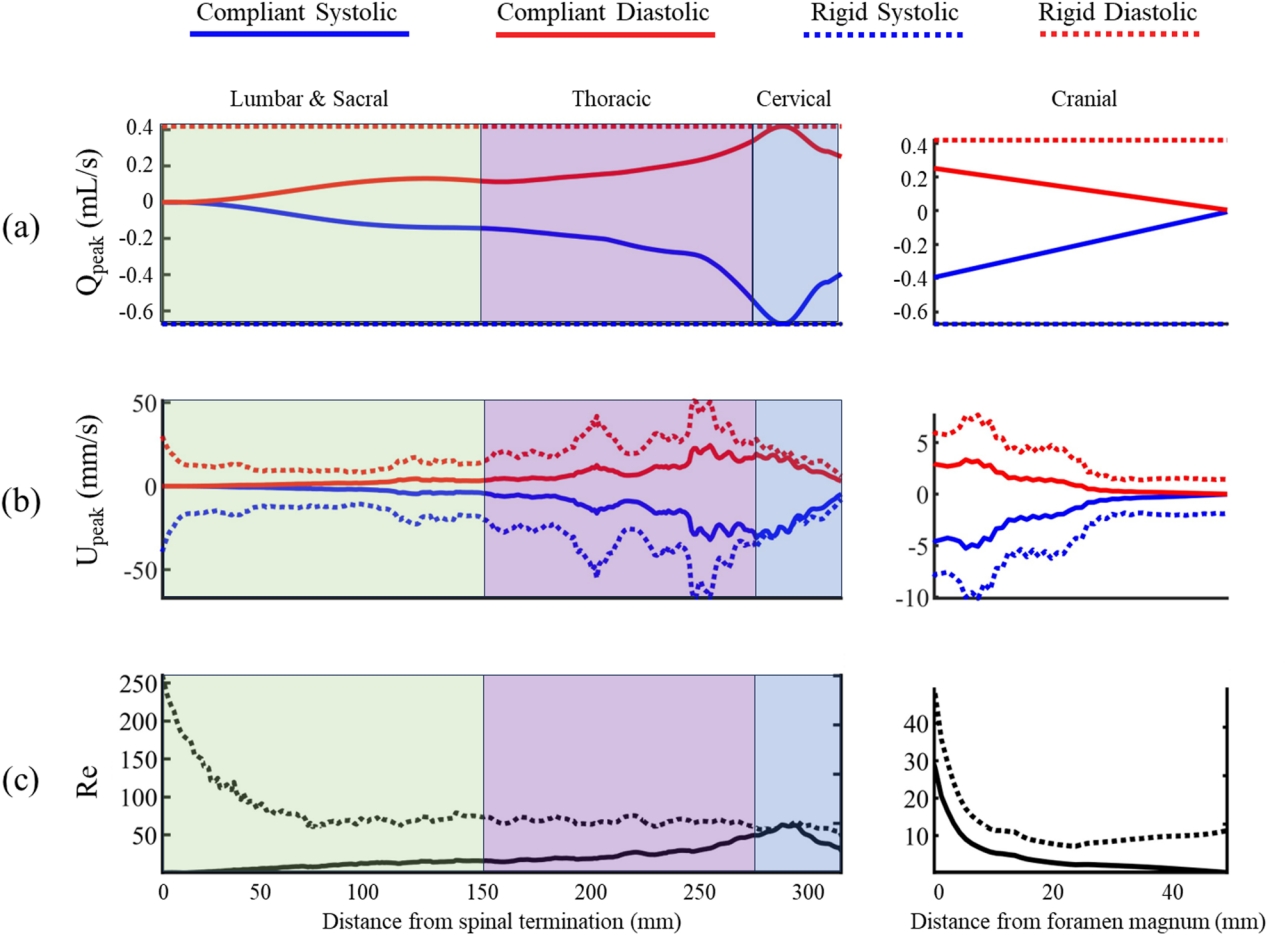
The distribution of hydrodynamic parameters for the compliant and rigid digital models across various anatomical regions including the dura mater, spinal cord, nerve roots and subarachnoid space along the spinal and cranial regions of a NHP. The parameters visualized include: (**a**) peak systolic and diastolic flowrates, (**b**) peak systolic and diastolic cerebrospinal fluid (CSF) velocities , and (**c**) Reynolds number (Re)

Introducing compliance to the digital model impacted solute dispersion (Figs. 5 and 6), CSF velocity profiles (Fig. 7), and CSF hydrodynamics (Fig. 8) to a great degree. For the compliant model, radial dural displacement ranged from a maximum of 120 μm in the cervical spine to a minimum of ~ 20 μm in the lumbar and cranial regions (Fig. 3b). The radial dural movement reproduced the MRI-derived oscillatory CSF flow along the spine with a maximum error of 2.8% at peak systole (Fig. 2a and c). Visual analysis of CSF velocity profiles in the compliant versus rigid digital model had similar features at the foramen magnum, C2/3, C5/6 (compare Fig. 7b versus 7c). However, at T4/5, T10/11, and L3/4 velocity profiles were different with the compliant model showing much smaller CSF velocities compared with the rigid digital model. Comparison of digital model velocity profiles to the in vivo phase-contrast MRI measurements showed that the compliant digital model had smaller peak velocities at C2/3 and T4/5 (compare Fig. 7a versus 7b).

Quantitatively, peak systolic and diastolic CSF flow rate (Q_peak_) and velocities (U_peak_) were greater in the rigid digital model compared with the compliant model throughout the spine and around the brain except in the cervical spine, where Q_peak_ and U_peak_ values were nearly identical (Fig. 8a and b). Similarly, Reynolds number (Fig. 8c) was impacted to a great degree by the addition of compliance to the digital model. In the rigid digital model, Reynolds number was ~ 100 throughout the spine, except in the lumbar spine where it increased up to 250 due to the reduction in subarachnoid space cross-sectional area at that region while Q_peak_ was consistent (Fig. 8a). In contrast, Reynolds number in the compliant digital model was smaller, with a maximum value of 61.8 along the spine and 29.1 around the brain due to flow reduction near model ends.

Visual analysis of tracer concentrations showed greater neuraxial dispersion in the rigid compared with compliant model (Fig. 5b1 and 5b2 versus 5c1 and 5c2). Tracer dispersion was notably slower in the compliant model where even 60 min post-injection, the tracer did not move beyond the T11 vertebral level. In contrast, at 60 min post-injection in the rigid model, the tracer moved to the cranial base. Quantitatively, at 60-minutes post-injection, ~ 90% of the injected dose remained in the lumbar spine for the compliant model compared with ~ 70% in the rigid model (Fig. 6a). The lack of dose dispersion in the compliant model resulted in a concentrated AUC near the injection site of 2.8 compared to 1.2%·hr for the rigid model (Fig. 6b). 60-minutes post injection, % injected dose to the intracranial compartment was 1.1% in the rigid model and ~ 0% for the compliant model (Fig. 6a).

## Discussion

Many treatments for CNS disorders require dosing via the CSF since they do not efficiently cross the blood brain barrier. However, CSF-dosing and drug distribution relies on a multitude of factors that are poorly understood and often overlooked when dosing parameters are determined. To ensure optimal treatment effect, it is imperative to ensure CSF drug dispersion around the brain and spinal cord. In vivo testing is time consuming and cost ineffective and therefore does not allow for optimization of many parameters. The presented NHP digital and bench-top CSF fluid dynamics modeling platform serves as a “digital twin” that mirrors in vivo CSF system physiology and anatomy relevant to intra-CSF solute dispersion. The platform was formulated using non-invasive MR imaging that can be collected on any animal species and, in principle, can be extended to incorporate patient-specific data to enable prediction of intra-CSF drug dispersion under various anatomical and flow conditions. Digital twin modeling may be used to refine intra-CSF drug delivery protocols for CNS-targeted therapies, improve device design features, supplement reliance on preclinical animal models and provide insight into interspecies dose scaling, all of which are key aspects to improve the effectiveness of CNS treatments.

### Uniqueness of the NHP model and allometry to humans

We present, to our knowledge, the first NHP-specific compliant digital model of the entire CSF system, encompassing both intracranial and spinal regions. The model integrates key intracranial spaces, CSF formation and efflux, detailed spinal cord nerve rootlet geometry and filum terminale, and a CSF waveform with both cardiac- and respiratory-induced flow. Total CSF volume is 16.2 mL (Table 2) using high-resolution MR imaging collected with 0.38 mm isotropic resolution. This total CSF volume is ~ 20% larger than previously reported by Sullivan et al. [40] using MR imaging collected at 0.7 mm in-plane and 2.0 mm slice thickness. The greater total CSF volume may be attributed to the 4.6 kg NHP imaged in our study compared to the 4.1 ± 0.5 kg NHPs imaged in Sullivan et al. Additionally, we found that much of the cortical CSF resides in spaces less than 0.5 mm thick. For the single NHP considered in the current study, 5.9 mL (70%, Table 2) of the intracranial CSF was present in the cortical region, pointing to the potential for underestimation of this small space at lower relative imaging resolution. In previous research conducted by our team, the spinal CSF for eight cynomolgus NHPs was found to occupy 7.4 mL, on average [30]. The single animal-specific spinal segmentation used in the current study was slightly above this average at 7.8 mL, representing 48% of the total CSF volume. Within the 16.2 mL total CSF volume, only 0.53 mL resides in the ventricular system. This ventricular volume is similar to the ~ 0.4 to 5 mL average reported by Alldritt et al. for 4-year-old NHPs [41].

Notable differences in NHP CSF system anatomy compared with humans were quantified in terms of anatomic CSF passageways and allometric scaling of the CNS tissues and fluids. Interestingly, the NHP 4th ventricle had a total of five openings leading to the basal cisterns of the brain (see Fig. 1e – 4th ventricle geometry). In the human, there are only three of these openings given by the foramina of Lushka (two lateral apertures) and foramen of Magendie (one median aperture). The percent of CSF volume in the ventricles compared to total CSF system was smaller in our NHP model compared to humans [42] (~ 3% versus ~ 10%). In the spinal subarachnoid space, idealized nerve root detail was added by scaling human nerve root geometry previously published by our group. This anatomy contributed less than 0.12 mL or 1% of the overall CNS volume but increased the total CNS surface area by 14% (22.7cm^2^) and by ~ 40% in the spinal canal. While these structures are relatively small by volume, they contribute a notable surface area for potential drug-tissue interaction. The ratio of NHP CNS tissue to CSF volume was 0.5:1 and 7.6:1 for the spine and intracranial region, respectively. In contrast, for the adult human female CSF system model previously published by our team [29], the ratio of human CNS tissue to CSF volume was 0.23:1 and 5.4:1 for the spine and intracranial region, respectively. The ratio of CSF between the spine and brain for the same cynomolgus and human models was 0.47:1 and 0.89:1. Finally, the brain volume to CSF contacting surface area in these models was found to be 0.36:1 and 0.91:1 for the NHP and human cases, respectively.

Allometric scaling analysis suggests that (a) CSF delivery and dosing protocols using overall CSF system volume may not scale directly from primates to humans and (b) the NHP represents a best-case scenario for LP-based intrathecal drug transport to the brain compared with humans. Due to the notably lower fraction of CSF in the spinal canal, drug transport in the NHP is likely to progress towards the brain more readily, notwithstanding the higher relative dosing volumes also typically used for CSF dosing in NHPs. Combined with a cortical surface area to volume ratio ~ 3 times higher than in humans, the targeting of intra-CSF delivery to the brain may be increased in NHP models. Overall, these results point to a need for digital and in vitro bench-top modeling to understand drug delivery dynamics and scaling between model species and humans. As such, detailed parametric studies with comparison across species and ages would be an important next step. It should be noted that many other physiologic and pharmacologic factors may also impact delivery success and care should be taken to evaluate the relative contribution of these individually.

### Digital model verification by bench-top and numerical independence studies

Digital model verification by real-world bench-top experiments and numerical sensitivity studies are important steps to help build confidence in model predictions. In combination, our results indicate that the real-world physics of CSF solute transport in the bench-top model were recapitulated in the digital model, adding confidence that the underlying assumptions and boundary conditions were applied correctly. In previous research conducted by our team, we performed numerical sensitivity studies to confirm time-step, mesh size, and flow cycle independence for similar adult human intra-CSF solute dispersion models [34, 43]. A key assumption was that molecular diffusion was an insignificant mode of mass transport and therefore was not included in the model physics. This assumption has been previously applied and verified by our team in human digital models [43, 44] and other research groups [39, 45].

Digital model solute dispersion predictions were verified by comparison to a novel 3D printed NHP CSF system bench-top model [15] (Fig. 1b) with identical flow and geometric boundary conditions. We chose to compare solute dispersion predictions for a rigid (non-compliant) CSF geometry because a compliant bench-top model would not allow precise control and certainty of CSF flow oscillations along the entire neuroaxis and within the ventricles of the brain for comparison to the digital model. Comparison of results showed strong agreement in terms of spatial-temporal tracer concentration (Fig. 5), percent of injected dose in the spine and brain (Fig. 6a), and AUC profile along the neuroaxis (Fig. 6b). Differences in regional percent of injected dose for the rigid digital and bench-top model did not exceed 5%. AUC profile for the bench-top model showed slightly greater tracer dispersion towards the brain compared with the rigid digital model (Fig. 6b). To further substantiate the comparison between the digital and bench-top model spatial-temporal tracer dispersion, we employed both regression and Bland-Altman plots showing R² = 0.88, Y-intercept = -0.02, slope = 1.07, and 95% confidence interval of less than 8.9% injected dose. The bench-top model geometric printing accuracy was previously validated by micro-CT scanning and confirmed for test-retest repeatability under a variety of injection protocols and devices [15]. The slight difference in digital model predictions from bench-top measurements may be attributed to 3D print accuracy limitations to replicate detailed lumbar filum terminale and nerve rootlets and tight CSF spaces around the cortical convexities (Fig. 1b and d).

### Inclusion of CSF system compliance decreases neuraxial solute dispersion

While CSF system compliance and the corresponding attenuation of cardiac-induced CSF oscillations along the neuroaxis has been extensively documented [26, 30, 46–52], little research has investigated to what degree compliance may impact neuraxial solute transport. Our findings indicate that inclusion of NHP-specific CSF system compliance decreased neuraxial solute dispersion along the spine to a great degree following a standard LP injection scenario. We emulated neuraxial compliance by applying a previously developed non-uniform moving boundary method that moved the dural surface to recapitulate the axial attenuation of CSF oscillations [14]. This method was previously validated to reproduce in vivo NHP-specific CSF oscillations along the spine with a maximum error of 2.8% at peak systole at six spinal locations. Here, we expanded the 3D model to include NHP-specific intracranial CSF and attenuate CSF oscillations around the brain up to the cranial apex at the superior sagittal sinus and included multiphase solute transport in the model solver.

As expected, addition of compliance to the model attenuated the velocity field in the lumbar region near the L3/4 injection site with a peak flow rate of ~ 0.1 mL/min compared to ~ 0.7 mL/min in the rigid digital model, while having no impact on CSF cross-sectional area (see Figs. 3b and 4b). In combination, smaller flow rate and weaker velocity field in the compliant model slowed tracer dispersion to a great degree with nearly all the solute remaining near the injection site even 1-hour after tracer injection (Fig. 6). Both the rigid and compliant digital model showed low tracer concentrations around the brain with 1.1% and ~ 0% ID, respectively at 1-hour following injection. While both numbers are relatively small fractions of the overall dose, it is worth noting that there is an order of magnitude between them. This factor could be significant when designing a dosing scheme.

Tracer dispersion during the 60 s tracer infusion procedure was nearly identical, with or without compliance included in the model (Fig. 5b-c). This period of agreement between the models is likely due to the dominant velocity field produced from the tracer injection of 1 mL/min compared with a 0.1 mL/min peak flowrate near the LP injection site due to lumbar CSF oscillations in the compliant model. While there is a substantial variance in the lumbar and thoracic regions with and without compliance, similar transport rates in the cervical region, specifically near C2/3, is expected since CSF oscillations are nearly identical in both models at that location due to specification of the C2/3 level flowrate across the entire rigid model neuroaxis (see methods). However, for the specified NHP-injection protocol (1 mL in 1 min at L3/4) the tracer did not reach the cervical region in the compliant model. Thus, different bolus volumes, rates, and injection locations are expected to have varying degrees of impact with or without CSF compliance included.

### Comparison of results to previous digital and laboratory bench-top models of CSF solute dispersion

Several studies have examined intra-CSF drug dispersion with and without spinal compliance, providing insights into how there different modeling approaches predict intrathecal drug dispersion. To our knowledge, one previous intra-CSF drug delivery computational model has been formulated for a NHP by Tangen et al. [13]. This model included NHP-specific spinal CSF compliance but lacked the intracranial CSF spaces and CSF formation rate, respiratory-induced CSF dynamics, dorsal and ventral spinal cord nerve rootlets, and filum terminale. The model predicted that an LP injection would remain primarily in the lumbar spine up to 1 h after infusion but did not directly compare the effect of a rigid versus compliant spine. In the present NHP model, we include anatomically detailed intracranial CSF spaces including extra-axial CSF with Sylvian cisterns, cisterna magna, lateral, third and 4th ventricles, CSF formation from the choroid plexus, aqueductal CSF oscillations, respiratory- and cardiac-induced CSF oscillations along the neuroaxis, and anatomically detailed spinal cord nerve rootlets and filum terminale. Burla et al. examined the impact of injection protocols on drug dispersion using a rigid bench-top NHP CSF system model and showed that solute dispersion was sensitive to bolus and flush volume and injection location along the spine [15]. Solute dispersion from the lumbar to cervical spine ranged from a few minutes to ~ 1 h, depending on protocol parameters. The present digital model has identical NHP geometry and flow boundary conditions as Burla et al. and highlights that inclusion of spinal compliance can alter solute dispersion to a great degree for a standard LP injection thereby preventing the solute from reaching the cervical spine in the same timeframe.

Several human intra-CSF drug delivery computational and bench-top models have been conducted and generally indicated a slow dispersion of LP injected solutes. Kutler et al., formulated a multiphase rigid human spinal model with simplified anatomy and demonstrated that the drug reached the cervical spine at ~ 60 min after LP injection [6]. Similarly, Khani et al. and Seiner et al., explored the effects of injection location, protocol, and physiology on CSF drug delivery using a rigid human digital and bench-top models [53] and also found that solute dispersion from the lumbar to cervical spine required ~ 60 min. Hsu et al., simulated spinal anesthetic dispersion in a human CSF flow model with compliant spine and predicted that an LP injected drug maximum concentration would occur ~ 30 min after injection at the C4 vertebral level [53]. These findings were subsequently verified and further investigated by Ayansiji et al. through construction of a novel compliant human spinal bench-top model [54]. Alaminos-Quesada et al. developed elegant leading-order analytic solutions aimed toward LP drug injection prediction within a compliant spine and showed that time-averaged Lagrangian motion of the fluid could be used to predict spinal solute dispersion with varying buoyancy while neglecting drug diffusivity [55] and verified analytic solutions against direct numerical simulations with strong agreement [56, 57]. However, these models did not aim to account for solute dispersion within the intracranial CSF. The authors noted that analytic results may not be as accurate for specific waveforms and minor anatomic structures, a factor also highlighted by numerical studies conducted by Wang et al. [58] and others [22, 59, 60].

### Comparison to in vivo

The presented NHP-specific digital model of CSF solute dispersion was formulated using animal specific in vivo MR imaging to specify the model geometric boundary conditions. However, the animal was not injected with a LP-based tracer, such as gadolinium or a radiolabeled tracer, to help directly validate model predictions on an animal-basis. Thus, here we compare model predictions against previously published solute transport findings in NHPs in the literature, as a proxy to help verify model agreement with in vivo dispersion characteristics. Overall, a key characteristic of the compliant digital model was that the LP injected solute had relatively little dispersion beyond the lumbar spine even 1 h after injection (Figs. 5c and 6a) suggesting that cerebral targets would be largely unreachable with a standard LP administration. These findings agree with in vivo NHP measurements published by several authors. Tangen et al. [13] reported that both 0.36 and 1.8 mL LP-based injections of ^64^Cu-DOTA in NHPs remained in the lower spine even 1 h after delivery. In contrast, Ohno et al. [61] conducted a NHP MR-study and imaged drug product spiked with gadolinium after 6 mL LP-based injections at a rate of 1 mL/min. Under these conditions, gadolinium dispersion was found to move relatively rapidly to the brain within just 15 min of injection and result in drug product leakage near the LP injection site in some animals [62]. The relatively rapid gadolinium dispersion in Ohno et al. [61] is expected since the NHP spine only contains ~ 7 mL of CSF [26, 30]. Thus, since the injection was rapid and large, dispersion of the solute was primarily by bulk fluid displacement. In the current study, we modeled a 1 mL LP injection at a rate of 1 mL/min. The mode of mass transport following LP bolus injection was advection due to relatively slow steady streaming flow characteristics along the spine. Although not directly comparable to the current study, Ballon et al. conducted a series of whole-body NHP imaging studies using a 1 mL bolus of I-124 labeled AAV injected to cisterna magna at a rate of 0.5 mL/min [62]. The drug product was found to disperse only ~ 1/2 way down the spine 1 h following injection, demonstrating a relatively slow movement, especially as it approached the lumbar spinal segment. In combination, these in vivo NHP findings aligned with our compliant model predictions showing that ~ 90% of the tracer remained in the lumbar spine up to 1 h following injection (Figs. 5c and 6).

### Limitations

This study aimed to predict the dispersion of a 1 mL tracer injected at 1 mL/min up to 1 hour following LP-based dosing, a scenario relevant for acute intrathecal drug applications in pre-clinical research studies. Future work could broaden this scope by delving into chronic intrathecal drug delivery, which involves the subcutaneous implantation of drug pumps and low-flow therapeutic agent dosing over extended periods (days, weeks, months) [34]. Such studies will necessitate consideration of the molecular and chemical kinetic properties of infused drugs, including their specific uptake into tissue via movement across the pia mater, transport along paravascular spaces, movement into the intracellular spaces, uptake into cells, and clearance by bulk flow pathways, cellular efflux, and metabolism. Additionally, infusion settings (bolus versus continuous), changes in CSF viscosity due to temperature of the injected bolus and catheter geometry can influence drug dispersion patterns. Laplacian smoothing was applied to the extra-axial CSF space segmentation. Future work could parametrically check how solute dispersion may be altered in smoothed versus non-smoothed geometries and with and without inclusion of arachnoid trabeculae microanatomy.

The moving boundary method applied in our study to mimic craniospinal compliance involved dura surface movement that was not directly observable from MRI images due to the displacements falling below the imaging threshold. Thus, our modeling strategy and findings are based on inferred deformations, whose exact magnitude and spatiotemporal distribution require experimental validation. Furthermore, to minimize the animal’s scanning time, we limited the cerebrospinal fluid phase-contrast MRI flow measurements to five regions of interest along the neuroaxis and inferred respiratory-induced CSF oscillations based on cardiac to respiratory power ratio quantified in humans along the spine and at the aqueduct of Sylvius. CSF oscillatory boundary conditions could be improved by application of real-time phase-contrast MRI in NHPs. The radial dura deformation approach used in this study represents an imposed wall motion strategy to emulate craniospinal compliance, rather than a direct modeling of compliance by pressure-volume relationship (fluid-structure interaction). This method mimics compliance by recapitulation its combined effect on CSF flow along the neuroaxis [13, 14]. Because our focus is on CSF solute dispersion, we believe this approach is useful as the CSF flow field would most directly impact solute dispersion. However, future work could investigate fully coupled fluid-structure models to capture the pressure-flow characteristics along the spine and integrate solute absorption at the CNS / CSF tissue interface to fully recapitulate drug dispersion deep into the brain and ultimately into cells. Our anatomic MR imaging protocol provided sub-millimeter voxel resolution that allowed for the reconstruction of large structures and some microanatomical structures, especially nerve roots, which are important for modeling geometry-induced dispersion [6]. Nevertheless, smaller microanatomical features, such as microvasculature within the CSF, arachnoid trabeculae, dorsal and dorsolateral septa, and denticulate ligaments, were excluded from the reconstruction due to their size falling below the MR image resolution threshold.

## Conclusion

Digital models are important tools that can be used to understand and optimize drug delivery within and across species. Here, we present a novel animal-specific NHP digital modelling platform for prediction of CSF system-wide solute dispersion using first principles with anatomical information and CSF flow measurements collected by non-invasive MRI. This model includes anatomically detailed geometry of the CSF system including fine anatomy such as the dorsal and ventral spinal cord nerve rootlets and physiologically relevant incorporation of cardiac- and respiratory-induced CSF oscillations and CSF formation/efflux. The model is verified numerically and validated against real-world solute dispersion measurements collected in a novel bench-top NHP analog and by comparison to in vivo solute dispersion characteristics reported in the literature. Our findings highlight the viability of employing computational methods to predict CSF solute dispersion and the important role of craniospinal compliance on model predictions. Computational methods capable of assessing and quantifying the distribution of intra-CSF delivered therapies may prove useful to reduce costs and accelerate therapeutic development pipelines. Future work should be aimed to improve models by honing physiologic boundary conditions and conducting extensive verification and validation studies to fully extract model predictive value.

## Electronic Supplementary Material

Below is the link to the electronic supplementary material.

Supplementary Material 1

## Data Availability

The data that support the findings of this study are available from the corresponding author upon reasonable request.

## Abbreviations

LP: Lumbar puncture
CFD: Computational fluid dynamics
CNS: Central nervous system
CSF: Cerebrospinal fluid
MRI: Magnetic resonance imaging

## References

[1] Pardridge WM. The blood-brain barrier: bottleneck in brain drug development. NeuroRx. 2005;2:3–14. 10.1602/neurorx.2.1.3 15717053 10.1602/neurorx.2.1.3 PMC539316

[2] Penn RD. Intrathecal Baclofen for spasticity of spinal origin: seven years of experience. J Neurosurg. 1992;77:236–40. 10.3171/jns.1992.77.2.0236 1625011 10.3171/jns.1992.77.2.0236

[3] Bennett G, et al. Evidence-based review of the literature on intrathecal delivery of pain medication. J Pain Symptom Manage. 2000;20:12–36. 10.1016/s0885-3924(00)00204-9. 10989255 10.1016/s0885-3924(00)00204-9

[4] Prager J, et al. Best practices for intrathecal drug delivery for pain. Neuromodulation. 2014;17:354–72. discussion 372. 10.1111/ner.12146 24446870 10.1111/ner.12146

[5] Duarte RV, Lambe T, Raphael JH, Eldabe S, Andronis L. Intrathecal drug delivery systems for the management of chronic non-cancer pain: protocol for a systematic review of economic evaluations. BMJ Open. 2016;6:e012285. 10.1136/bmjopen-2016-012285. 27421298 10.1136/bmjopen-2016-012285 PMC4964247

[6] Kuttler A, et al. Understanding pharmacokinetics using realistic computational models of fluid dynamics: biosimulation of drug distribution within the CSF space for intrathecal drugs. J Pharmacokinet Pharmacodyn. 2010;37:629–44. 10.1007/s10928-010-9184-y. 21132572 10.1007/s10928-010-9184-y PMC3005107

[7] Sadekar SS, et al. Translational approaches for brain delivery of biologics via cerebrospinal fluid. Clin Pharmacol Ther. 2022;111:826–34. 10.1002/cpt.2531. 35064573 10.1002/cpt.2531 PMC9305158

[8] Hunt MA, Hunt SAC, Edinger K, Steinauer J, Yaksh TL. Refinement of intrathecal catheter design to enhance neuraxial distribution. J Neurosci Methods. 2024;402:110006. 10.1016/j.jneumeth.2023.110006. 37967672 10.1016/j.jneumeth.2023.110006

[9] Daouk J, Bouzerar R, Baledent O. Heart rate and respiration influence on macroscopic blood and CSF flows. Acta Radiol. 2017;58:977–82. 10.1177/0284185116676655. 28273732 10.1177/0284185116676655

[10] Stoquart-ElSankari S, et al. Aging effects on cerebral blood and cerebrospinal fluid flows. J Cereb Blood Flow Metab. 2007;27:1563–72. 10.1038/sj.jcbfm.9600462. 17311079 10.1038/sj.jcbfm.9600462

[11] Knox EG, Aburto MR, Clarke G, Cryan JF, O’Driscoll CM. The blood-brain barrier in aging and neurodegeneration. Mol Psychiatry. 2022;27:2659–73. 10.1038/s41380-022-01511-z. 35361905 10.1038/s41380-022-01511-z PMC9156404

[12] Ackley D, et al. FDA and industry collaboration: identifying opportunities to further reduce reliance on nonhuman primates for nonclinical safety evaluations. Regul Toxicol Pharmacol. 2023;138:105327. 10.1016/j.yrtph.2022.105327. 36586472 10.1016/j.yrtph.2022.105327

[13] Tangen K, et al. In vivo intrathecal tracer dispersion in cynomolgus monkey validates wide biodistribution along neuraxis. IEEE Trans Biomed Eng. 2020;67:1122–32. 10.1109/TBME.2019.2930451. 31352328 10.1109/TBME.2019.2930451

[14] Khani M, et al. Nonuniform moving boundary method for computational fluid dynamics simulation of intrathecal cerebrospinal flow distribution in a cynomolgus monkey. J Biomech Eng. 2017;139. 10.1115/1.4036608. 10.1115/1.4036608 PMC5467026 28462417

[15] Burla GKR, Shrestha D, Bowen M, Horvath JD, Martin BA. Evaluating the effect of injection protocols on intrathecal solute dispersion in non-human primates: an in vitro study using a cynomolgus cerebrospinal fluid system. Fluids Barriers CNS. 2024;21:61. 10.1186/s12987-024-00556-2. 39061067 10.1186/s12987-024-00556-2 PMC11282645

[16] Linninger AA, Somayaji MR, Erickson T, Guo X, Penn R. D. Computational methods for predicting drug transport in anisotropic and heterogeneous brain tissue. J Biomech. 2008;41:2176–87. 10.1016/j.jbiomech.2008.04.025. 18550067 10.1016/j.jbiomech.2008.04.025

[17] Pizzichelli G, et al. Numerical study of intrathecal drug delivery to a permeable spinal cord: effect of catheter position and angle. Comput Methods Biomech Biomed Engin. 2017;20:1599–608. 10.1080/10255842.2017.1393805. 29119834 10.1080/10255842.2017.1393805

[18] Keith Sharp M, Carare RO, Martin BA. Dispersion in porous media in oscillatory flow between flat plates: applications to intrathecal, periarterial and paraarterial solute transport in the central nervous system. Fluids Barriers CNS. 2019;16:13. 10.1186/s12987-019-0132-y. 31056079 10.1186/s12987-019-0132-y PMC6512764

[19] Hsu Y, Harris TJ, Hettiarachchi HDM, Penn R, Linninger AA. Three dimensional simulation and experimental investigation of intrathecal drug delivery in the spinal canal and the brain. 21st Eur Symp Comput Aided Process Eng. 2011;29:1525–9.

[20] Hsu Y, Hettiarachchi HD, Zhu DC, Linninger AA. The frequency and magnitude of cerebrospinal fluid pulsations influence intrathecal drug distribution: key factors for interpatient variability. Anesth Analg. 2012;115:879–879. 10.1213/ANE.0b013e3182536211 22523420

[21] Tangen K, et al. Clearance of subarachnoid hemorrhage from the cerebrospinal fluid in computational and in vitro models. Ann Biomed Eng. 2016. 10.1007/s10439-016-1681-8. 27384938 10.1007/s10439-016-1681-8

[22] Tangen KM, Hsu Y, Zhu DC, Linninger AA. CNS wide simulation of flow resistance and drug transport due to spinal microanatomy. J Biomech. 2015;48:2144–54. 10.1016/j.jbiomech.2015.02.018. 25888012 10.1016/j.jbiomech.2015.02.018

[23] Haga PT, et al. A numerical investigation of intrathecal isobaric drug dispersion within the cervical subarachnoid space. PLoS ONE. 2017;12:e0173680. 10.1371/journal.pone.0173680. 28296953 10.1371/journal.pone.0173680 PMC5351861

[24] Pizzichelli G, et al. Numerical study of intrathecal drug delivery to a permeable spinal cord: effect of catheter position and angle. Comput Methods Biomech Biomed Engin. 2017;1–10. 10.1080/10255842.2017.1393805. 10.1080/10255842.2017.1393805 29119834

[25] Khani M, et al. Anthropomorphic model of intrathecal cerebrospinal fluid dynamics within the spinal subarachnoid space: spinal cord nerve roots increase Steady-Streaming. J Biomech Eng. 2018;140. 10.1115/1.4040401. 10.1115/1.4040401 PMC6056198 30003260

[26] Khani M, et al. Intrathecal catheter implantation decreases cerebrospinal fluid dynamics in cynomolgus monkeys. PLoS ONE. 2020;15:e0244090. 10.1371/journal.pone.0244090. 33378399 10.1371/journal.pone.0244090 PMC7773283

[27] Pardo ID, et al. Technical guide for nervous system sampling of the cynomolgus monkey for general toxicity studies. Toxicol Pathol. 2012;40:624–36. 10.1177/0192623311436180. 22317925 10.1177/0192623311436180

[28] Nikolenko VN. [Length of the spinal dural sac, sex differences and correlation with the length of the spinal cord and vertebral column in adults]. Arkh Anat Gistol Embriol. 1985;88:23–5. 4051760

[29] Sass LR, et al. A 3D subject-specific model of the spinal subarachnoid space with anatomically realistic ventral and dorsal spinal cord nerve rootlets. Fluids Barriers CNS. 2017;14:36. 10.1186/s12987-017-0085-y. 29258534 10.1186/s12987-017-0085-y PMC5738087

[30] Khani M, et al. Characterization of intrathecal cerebrospinal fluid geometry and dynamics in cynomolgus monkeys (macaca fascicularis) by magnetic resonance imaging. PLoS ONE. 2019;14:e0212239. 10.1371/journal.pone.0212239. 30811449 10.1371/journal.pone.0212239 PMC6392269

[31] Khani M, et al. Nonuniform moving boundary method for computational fluid dynamics simulation of intrathecal cerebrospinal flow distribution in a cynomolgus monkey. J Biomech Eng. 2017;139:0810051–08100512. 10.1115/1.4036608. 28462417 10.1115/1.4036608 PMC5467026

[32] Yildiz S, et al. Quantifying the influence of respiration and cardiac pulsations on cerebrospinal fluid dynamics using real-time phase-contrast MRI. J Magn Reson Imaging. 2017;46:431–9. 10.1002/jmri.25591. 28152239 10.1002/jmri.25591

[33] Lester McCully CM, et al. Flow rate and apparent volume of cerebrospinal fluid in rhesus macaques (Macaca mulatta) based on the pharmacokinetics of intrathecally administered inulin. Comp Med. 2020;70:526–31. 10.30802/AALAS-CM-99-990010. 33046181 10.30802/AALAS-CM-99-990010 PMC7754203

[34] Khani M, et al. Human in Silico trials for parametric computational fluid dynamics investigation of cerebrospinal fluid drug delivery: impact of injection location, injection protocol, and physiology. Fluids Barriers CNS. 2022;19:8. 10.1186/s12987-022-00304-4. 35090516 10.1186/s12987-022-00304-4 PMC8796513

[35] Watts R, Steinklein JM, Waldman L, Zhou X, Filippi CG. Measuring glymphatic flow in man using quantitative contrast-enhanced MRI. AJNR Am J Neuroradiol. 2019;40:648–51. 10.3174/ajnr.A5931 30679221 10.3174/ajnr.A5931 PMC7048502

[36] Lester McCully C, et al. Plasma and cerebrospinal fluid pharmacokinetics of the DNA methyltransferase inhibitor, 5-azacytidine, alone and with inulin, in nonhuman primate models. Neurooncol Adv. 2020;2:vdaa005. 10.1093/noajnl/vdaa005. 32309806 10.1093/noajnl/vdaa005 PMC7146732

[37] Lui AC, Polis TZ, Cicutti NJ. Densities of cerebrospinal fluid and spinal anaesthetic solutions in surgical patients at body temperature. Can J Anaesth. 1998;45:297–303. 10.1007/BF03012018. 9597201 10.1007/BF03012018

[38] Seiner A, et al. Investigation of human intrathecal solute transport dynamics using a novel in vitro cerebrospinal fluid system analog. Front Neuroimaging. 2022;1. 10.3389/fnimg.2022.879098. 10.3389/fnimg.2022.879098 PMC10406265 37555174

[39] Tangen K, et al. Clearance of subarachnoid hemorrhage from the cerebrospinal fluid in computational and in vitro models. Ann Biomed Eng. 2016;44:3478–94. 10.1007/s10439-016-1681-8. 27384938 10.1007/s10439-016-1681-8

[40] Sullivan JM, et al. Convective forces increase rostral delivery of intrathecal radiotracers and antisense oligonucleotides in the cynomolgus monkey nervous system. J Transl Med. 2020;18:309. 10.1186/s12967-020-02461-2. 32771027 10.1186/s12967-020-02461-2 PMC7414676

[41] Alldritt S, et al. Brain charts for the rhesus macaque lifespan. BioRxiv. 2024. 10.1101/2024.08.28.610193. 39257737 10.1101/2024.08.28.610193 PMC11383706

[42] Levi Chazen J, et al. Automated segmentation of MR imaging to determine normative central nervous system cerebrospinal fluid volumes in healthy volunteers. Clin Imaging. 2017;43:132–5. 10.1016/j.clinimag.2017.02.007. 28314198 10.1016/j.clinimag.2017.02.007

[43] Khani M, et al. Impact of neurapheresis system on intrathecal cerebrospinal fluid dynamics: A computational fluid dynamics study. J Biomech Eng. 2020;142. 10.1115/1.4044308. 10.1115/1.4044308 PMC7104775 31343659

[44] Khani M, et al. In vitro and numerical simulation of blood removal from cerebrospinal fluid: comparison of lumbar drain to neurapheresis therapy. Fluids Barriers CNS. 2020;17(23). 10.1186/s12987-020-00185-5. 10.1186/s12987-020-00185-5 PMC7077023 32178689

[45] Kurtcuoglu V, Soellinger M, Summers P, Poulikakos D, Boesiger P. Mixing and modes of mass transfer in the third cerebral ventricle: a computational analysis. J Biomech Eng. 2007;129:695–702. 10.1115/1.2768376. 17887895 10.1115/1.2768376

[46] Kalata W, et al. MR measurement of cerebrospinal fluid velocity wave speed in the spinal Canal. IEEE Trans Biomed Eng. 2009;56:1765–8. 10.1109/TBME.2008.2011647. 19174343 10.1109/TBME.2008.2011647

[47] Yiallourou TI, et al. Comparison of 4D phase-contrast MRI flow measurements to computational fluid dynamics simulations of cerebrospinal fluid motion in the cervical spine. PLoS ONE. 2012;7:e52284. 10.1371/journal.pone.0052284. 23284970 10.1371/journal.pone.0052284 PMC3528759

[48] Bunck AC, et al. Magnetic resonance 4D flow analysis of cerebrospinal fluid dynamics in Chiari I malformation with and without Syringomyelia. Eur Radiol. 2012;22:1860–70. 10.1007/s00330-012-2457-7. 22569996 10.1007/s00330-012-2457-7

[49] Bunck AC, et al. Magnetic resonance 4D flow characteristics of cerebrospinal fluid at the craniocervical junction and the cervical spinal Canal. Eur Radiol. 2011;21:1788–96. 10.1007/s00330-011-2105-7. 21404133 10.1007/s00330-011-2105-7

[50] Gutierrez-Montes C, et al. Effect of normal breathing on the movement of CSF in the spinal subarachnoid space. AJNR Am J Neuroradiol. 2022;43:1369–74. 10.3174/ajnr.A7603. 35981761 10.3174/ajnr.A7603 PMC9451622

[51] Sincomb S, et al. A one-dimensional model for the pulsating flow of cerebrospinal fluid in the spinal Canal. J Fluid Mech. 2022;939. 10.1017/jfm.2022.215. 10.1017/jfm.2022.215 PMC9635490 36337071

[52] Coenen W, et al. Subject-Specific studies of CSF bulk flow patterns in the spinal canal: implications for the dispersion of solute particles in intrathecal drug delivery. AJNR Am J Neuroradiol. 2019;40:1242–9. 10.3174/ajnr.A6097. 31196863 10.3174/ajnr.A6097 PMC7048533

[53] Hsu Y, Hettiarachchi HD, Zhu DC, Linninger AA. The frequency and magnitude of cerebrospinal fluid pulsations influence intrathecal drug distribution: key factors for interpatient variability. Anesth Analg. 2012;115:386–94. 10.1213/ANE.0b013e3182536211. 22523420 10.1213/ANE.0b013e3182536211

[54] Ayansiji AO, et al. Determination of spinal tracer dispersion after intrathecal injection in a deformable CNS model. Front Physiol. 2023;14:1244016. 10.3389/fphys.2023.1244016. 37817986 10.3389/fphys.2023.1244016 PMC10561273

[55] Alaminos-Quesada J, Coenen W, Gutierrez-Montes C, Sanchez AL. Buoyancy-modulated lagrangian drift in wavy-walled vertical channels as a model problem to understand drug dispersion in the spinal Canal. J Fluid Mech. 2022;949. 10.1017/jfm.2022.799. 10.1017/jfm.2022.799 PMC10338005 37441053

[56] Alaminos-Quesada J, Gutierrez-Montes C, Coenen W, Sanchez AL. Stationary flow driven by non-sinusoidal time-periodic pressure gradients in wavy-walled channels. Appl Math Model. 2023;122:693–705. 10.1016/j.apm.2023.06.013. 37485297 10.1016/j.apm.2023.06.013 PMC10359023

[57] Alaminos-Quesada J, Gutierrez-Montes C, Coenen W, Sanchez AL. Effects of buoyancy on the dispersion of drugs released intrathecally in the spinal Canal. J Fluid Mech. 2024;985. 10.1017/jfm.2024.297. 10.1017/jfm.2024.297 PMC11108058 38774672

[58] Wang Z, Majidi M, Li C, Ardekani A. Numerical study of the effects of minor structures and mean velocity fields in the cerebrospinal fluid flow. Fluids Barriers CNS. 2024;21:102. 10.1186/s12987-024-00604-x. 39696350 10.1186/s12987-024-00604-x PMC11657969

[59] Heidari Pahlavian S, et al. The impact of spinal cord nerve roots and denticulate ligaments on cerebrospinal fluid dynamics in the cervical spine. PLoS ONE. 2014;9:e91888. 10.1371/journal.pone.0091888. 24710111 10.1371/journal.pone.0091888 PMC3977950

[60] Stockman HW. Effect of anatomical fine structure on the dispersion of solutes in the spinal subarachnoid space. J Biomech Eng. 2007;129:666–75. 10.1115/1.2768112. 17887892 10.1115/1.2768112

[61] Ohno K, et al. Kinetics and MR-Based monitoring of AAV9 vector delivery into cerebrospinal fluid of nonhuman primates. Mol Ther Methods Clin Dev. 2019;13:47–54. 10.1016/j.omtm.2018.12.001. 30666308 10.1016/j.omtm.2018.12.001 PMC6330508

[62] Grunert P, et al. Assessment of intervertebral disc degeneration based on quantitative magnetic resonance imaging analysis: an in vivo study. Spine (Phila Pa 1976). 2014;39:E369–378. 10.1097/BRS.0000000000000194. 24384655 10.1097/BRS.0000000000000194 PMC4486300

[63] Ringstad G, Eide PK. Cerebrospinal fluid tracer efflux to parasagittal dura in humans. Nat Commun. 2020;11:354. 10.1038/s41467-019-14195-x. 31953399 10.1038/s41467-019-14195-x PMC6969040

