# Supplementary material for: A 3D non-human primate digital model for pharmacokinetic prediction of intra-cerebrospinal fluid drug neuraxial dispersion
Source: Fluids Barriers CNS. 2025 Nov 4;22:111. doi: 10.1186/s12987-025-00723-z (PMC12584216; doi:10.1186/s12987-025-00723-z)
Supplement: Supplementary file 1 — Supplementary Material 1 [file 12987_2025_723_MOESM1_ESM.docx]

**Supplementary Table S1 – Literature review of intra-CSF dosing parameters.**

| Parameter family | Description | Species / model | Key references |
| --- | --- | --- | --- |
| Bolus design | Volume, viscosity, density, temperature | Human CFD; NHP in‑vivo | Kutler 2010;^6^ Tangen 2015^22^  Khani et al. 2022^34^ |
| Flush design | Volume, rate, orientation | NHP bench‑top | Burla 2024^15^ |
| Device factors | Needle gauge, side‑hole pattern, catheter tip | Large‑animal cadaver | Hunt 2024^8^ |
| Physiology | CSF formation/clearance, cardiac & respiratory rates | Human MRI; NHP MRI | Daouk 2017^9^; Yildiz 2017^32^ |
| Posture & gravity | Head‑down tilt, rotation maneuvers | Human MRI | Coenen 2019^52^ |
| Compliance & ICP | Craniospinal compliance, pressure spikes | Human infusion tests | Eide 2017^63^ |
| Anatomy | Nerve roots, filum, ventricles | Human & NHP CFD | Heidari 2014^59^; Wang 2024^58^ |
| Drug properties | Size, buoyancy, binding kinetics | Human & rodent PK | Sadekar 2022^7^ |
| Interspecies scaling | Allometric factors | NHP → Human | Sullivan 2020^40^ |
